# Using Sequence-Specific Chemical and Structural Properties of DNA to Predict Transcription Factor Binding Sites

**DOI:** 10.1371/journal.pcbi.1001007

**Published:** 2010-11-18

**Authors:** Amy L. Bauer, William S. Hlavacek, Pat J. Unkefer, Fangping Mu

**Affiliations:** 1 Theoretical Biology and Biophysics Group, Theoretical Division, Los Alamos National Laboratory, Los Alamos, New Mexico, United States of America; 2 National Stable Isotope Resource, Bioscience Division, Los Alamos National Laboratory, Los Alamos, New Mexico, United States of America; University of California, Davis, United States of America

## Abstract

An important step in understanding gene regulation is to identify the DNA binding sites recognized by each transcription factor (TF). Conventional approaches to prediction of TF binding sites involve the definition of consensus sequences or position-specific weight matrices and rely on statistical analysis of DNA sequences of known binding sites. Here, we present a method called SiteSleuth in which DNA structure prediction, computational chemistry, and machine learning are applied to develop models for TF binding sites. In this approach, binary classifiers are trained to discriminate between true and false binding sites based on the sequence-specific chemical and structural features of DNA. These features are determined via molecular dynamics calculations in which we consider each base in different local neighborhoods. For each of 54 TFs in *Escherichia coli*, for which at least five DNA binding sites are documented in RegulonDB, the TF binding sites and portions of the non-coding genome sequence are mapped to feature vectors and used in training. According to cross-validation analysis and a comparison of computational predictions against ChIP-chip data available for the TF Fis, SiteSleuth outperforms three conventional approaches: Match, MATRIX SEARCH, and the method of Berg and von Hippel. SiteSleuth also outperforms QPMEME, a method similar to SiteSleuth in that it involves a learning algorithm. The main advantage of SiteSleuth is a lower false positive rate.

## Author Summary

An important step in characterizing the genetic regulatory network of a cell is to identify the DNA binding sites recognized by each transcription factor (TF) protein encoded in the genome. Current computational approaches to TF binding site prediction rely exclusively on DNA sequence analysis. In this manuscript, we present a novel method called SiteSleuth, in which classifiers are trained to discriminate between true and false binding sites based on the sequence-specific chemical and structural features of DNA. According to cross-validation analysis and a comparison of computational predictions against ChIP-chip data available for the TF Fis, SiteSleuth predicts fewer estimated false positives than any of four other methods considered. A better understanding of gene regulation, which plays a central role in cellular responses to environmental changes, is a key to manipulating cellular behavior for a variety of useful purposes, as in metabolic engineering applications.

## Introduction

An important step in characterizing the genetic regulatory network of a cell is to identify the DNA binding sites recognized by each transcription factor (TF) protein encoded in the genome. A TF typically activates and/or represses genes by associating with specific DNA sequences. Although other factors, such as metabolite binding partners and protein-protein interactions (for example, between a TF and RNA polymerase or a second TF), can affect gene expression [1], it is important to identify the sequences directly recognized by TFs to the best of our ability to understand which genes are controlled by which TFs. A better understanding of gene regulation, which plays a central role in cellular responses to environmental changes, is a key to manipulating cellular behavior for a variety of useful purposes, as in metabolic engineering applications [2].

A number of computational methods have been developed for predicting TF binding sites given a set of known binding sites [3]–[10]. Commonly used methods involve the definition of a consensus sequence or the construction of a position-specific weight matrix (PWM), where DNA binding sites are represented as letter sequences from the alphabet {A, T, C, G}. More sophisticated approaches further constrain the set of potential binding sites for a given TF by considering, in addition to PWMs, the contribution of each nucleotide to the free energy of protein binding [3] and additional biologically relevant information, such as nucleotide correlation between different positions of a sequence [8] or sequence-specific binding energies [6]. Perhaps not as widely used as sequence analysis, the idea of employing structural data for predicting TF binding sites has been considered [11]–[15]. Most of these methods use protein-DNA structures rather than DNA by itself.

Acquiring training sets large enough to be useful is problematic for even well-studied TFs, for which only small sets of known binding sites (on the order of 10 sites) are typically available [8]. New high-throughput technologies have been used to identify large numbers of binding sites for particular TFs [16]–[18], but there remains a need for methods that predict TF binding sites given a small number of positive examples. Such methods can be used, for example, to complement analysis of high-throughput data. Binding sites detected by high-throughput *in vitro* methods, such as protein-binding microarrays [16], can be compared with predicted binding sites to prioritize studies aimed at confirming the importance of sites in regulating gene expression *in vivo*.

The fine three-dimensional (3D) structure of DNA is sequence dependent and TF-DNA interactions depend on various physicochemical parameters, such as contacts between nucleotides and amino acid residues and base pair geometry [19]. These parameters are not accounted for by conventional methods for predicting TF binding sites, which rely on sequence information alone. Letter representations of DNA sequences do not capture the biophysics underlying TF-DNA interactions. Given that a TF does not read off letters from a DNA sequence, but interacts with a particular sequence because of its chemical and structural features, we hypothesized that better predictions of TF binding sites might be generated by explicitly accounting for these features in an algorithm for predicting TF binding sites.

The mechanisms by which TFs recognize DNA sequences can be divided into two classes: indirect readout and direct readout [19]. For indirect readout, a TF recognizes a DNA sequence via the conformation of the sequence, which is determined by the local geometry of base pair steps, the distortion flexibility of the DNA sequence, and (water-mediated) protein-DNA interactions [20], [21]. For direct readout, a TF recognizes a DNA sequence through direct contacts between specific bases of the sequence and amino acid residues of the TF [22], [23]. These two classes of recognition mechanisms are not mutually exclusive.

In this study, we introduce a method, SiteSleuth, for predicting TF binding sites on the basis of sequence-dependent structural and chemical features of short DNA sequences. By using molecular dynamics (MD) methods to calculate these features, we can map a set of known or potential binding sites for a given TF to vectors of structural and chemical features. We use features of positive and negative examples of TF binding sites to train a support vector machine (SVM) to discriminate between true and false binding sites. Negative examples are derived from randomly selected non-coding DNA sequences. Positive examples are taken from RegulonDB [24], which collects information about TFs in *Escherichia coli*. Classifiers for *E. coli* TFs developed through the SiteSleuth approach are evaluated by cross validation, and the classifier for Fis is tested against chromatin immunoprecipitation (ChIP)-chip assays of Fis binding sites [17]. Combining ChIP with microarray technology, ChIP-chip assays provide information about DNA-protein binding *in vivo* on a genome-wide scale [25]. We also evaluate the performance of SiteSleuth against four other computational methods: the method of Berg and von Hippel (BvH) [3], MATRIX SEARCH [5], Match [7], and QPMEME [6]. The BvH, MATRIX SEARCH, and Match methods rely on the PWM approach to capture TF preferences for binding sites. The QPMEME method is similar to SiteSleuth in that it employs a learning algorithm. In the case of Fis, we show that SiteSleuth generates significantly fewer estimated false positives and provides higher prediction accuracy than the other computational approaches.

## Methods

Our supervised learning approach, which we call SiteSleuth, involves training a linear SVM classifier to distinguish TF binding sites documented in RegulonDB from randomly selected non-coding DNA sequences, which we take to represent negative examples of TF binding sites.

Briefly, a linear SVM classifier is an (*n−1*)-dimensional hyperplane in a *n*-dimensional feature space that maximally separates positive and negative training examples, if possible. When the training data can be separated by a hyperplane (**w** ^T^ **x**+d = 0), two parallel hyperplanes, given by **w** ^T^ **x**+d = ±1, mark the boundaries that maximize the distance between positive and negative examples (2/∥**w**∥). The quantity **x** is a vector of features, **w** is a weight vector of length *n*, and ∥**w**∥^2^ = **w** ^T^ **w**. A larger distance 2/∥**w**∥ results in a lower generalization error of the classifier. Positive examples lie on the positive side of **w** ^T^ **x**+d = 1 and negative examples lie on the negative side of **w** ^T^ **x**+d = −1. The parameters **w** and d of a classifier are determined by solving an optimization problem [26].

On the other hand, if no hyperplane exists that completely separates positive and negative examples, which is generally the case here, **w** and d can be determined using a soft margin method [26], which finds a hyperplane that achieves the largest separation distance possible with the smallest error penalty imposed by non-zero slack variables, *ζ_k_* (*k* = 1,…, *N*), where *N* is the number of training examples, both positive and negative. The soft margin method trades off separation and misclassification. Another way to deal with training examples that cannot be fully separated is to use a nonlinear SVM. Because the computational cost of using a nonlinear SVM for our purposes would be expensive, we opted to use a linear SVM with slack variables. The method of finding classifier parameters is briefly described below.

### Classifier training

Let us use **X** = {**x_1_**,…, **x_N_**} to represent the set of training data, where **x** *_k_* (*k* = 1,…, *N*) is a real-valued *n*-dimensional feature vector that characterizes the *k* ^th^ training example and *n* is the number of features considered. The features considered are described below. Given input **x** *_k_* and scalar output y*_k_* = {−1,1}, which identifies a training example as a positive or negative example of a binding site, classifier training produces an (*n−1*)-dimensional hyperplane in the space of features that satisfies the equation **w** ^T^ **x**+d = 0 and a set of linear inequality constraints, each involving a slack variable. The parameters **w** and d and the slack variables ξ*_k_* (*k* = 1,…, *N*) are found by solving the minimization problem
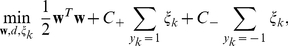
(1a)subject to the following constraints
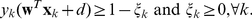
(1b)where *C*_+_ and *C*_−_ are penalty parameters [27]. These parameters are introduced to balance the contributions of negative and positive training examples to the objective function (Eq. 1a), as we typically have available many more negative examples than positive examples. The penalty parameters are determined for each TF via a grid search over ranges of *C_−_* and *C_+_* values as part of a 3-fold cross-validation procedure for each classifier.

In 3-fold cross validation, we randomly divide the training set into three subsets of roughly equal size. One subset is then used to test the accuracy of the classifier trained on the remaining two subsets until each subset has been used in testing. We used the *F*-measure to assess accuracy. The *F*-measure is the harmonic mean of precision (*p*) and recall (*r*):
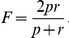
Precision is the fraction of predicted binding sites that are actually binding sites and recall is the fraction of actual binding sites predicted to be binding sites:
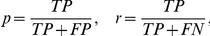
where *TP*, *FP*, and *FN* represent true positives, false positives and false negatives from 3-fold cross validation. To find values of *C_−_* and *C_+_* that maximize the *F*-measure, we first performed a coarse grid search over the following grid points: *C_−_* = [2^−5^, 2^−3^, …, 2^15^] and *C_+_* = [2^−5^, 2^−3^,…, 2^15^]. We then performed fine grid searches using progressively smaller grid spacing (2, 2^0.5^, 2^0.125^,…) around the best *C_−_* and *C_+_* values found in the coarse grid search.

### SiteSleuth prediction

Once trained, a classifier for a TF, taken to recognize binding sites of length *L*, is used for prediction as follows. The classifier is used to scan an organism's genome for binding sites of length *L*. Given a feature vector **x** *_m_* for a potential binding site *m*, we calculate the quantity **w** ^T^ **x** *_m_*+d. The decision function of the classifier is the sign of **w** ^T^ **x** *_m_*+d. Thus, if the sign of this quantity is positive, then site *m* is predicted to be a TF binding site. Conversely, a negative quantity indicates that *m* is not a binding site. This step is repeated for all non-coding sequences in the *E. coli* genome of length *L*. The length *L* was chosen for each TF based on information in RegulonDB [24].

### Structural and chemical features

Structural and chemical features of short DNA sequences were defined based on the predicted 3D structures of these DNA sequences, which were determined via MD simulations. MD simulations of solvated nucleic acids have been performed for almost three decades [28], [29]. Simulations of DNA oligomers have been studied systematically and results have been discussed in multiple publications [30]–[32]. Our approach is similar to that used in Refs. [30]–[32] and is described below. Because the available experimental data are incomplete (i.e., structures are unavailable for all 4-mers, at least in the Nucleic Acid Database [33]) and available structures have been determined under various experimental conditions (e.g., free or bound to protein), we used simulated structures rather than experimentally determined structures for determining structural and chemical features. Predicted structures were obtained for a common condition in a uniform manner.

#### Structural features

For an indirect readout mechanism, a TF recognizes DNA conformation, the local structure of DNA. To calculate structural features of base pairs, we considered all possible 3-mers and 4-mers of DNA. Each of the 3-mers (4-mers) was embedded within flanking GC nucleotide pairs to generate 7-mers (8-mers). Flanking nucleotide pairs are added to eliminate edge effects of 3-mers or 4-mers of DNA. We chose to cap both ends with GC nucleotide pairs, which is a common choice for reasons of rigidity and symmetry [30]–[32]. For each 7-mer or 8-mer, its initial 3D structure was generated using the 3DNA software [34]. The structure produced by 3DNA is based on the Watson and Crick DNA structure. The 3D DNA fragments were solvated and ionized to balance the negative charges of the DNA backbone. Final structures were obtained using the NAMD software tool [35] for MD simulations with the CHARMM27 force field parameters [36]. Other MD software packages could also have been used to obtain 3D DNA structures, but NAMD was a convenient choice for us because of our familiarity with this package. For each NAMD simulation, we performed 3 picoseconds (ps) of minimization, 7 ps of heating to 300 K, 30 ps of relaxation, and 50 ps of equilibration, followed by 1 nanosecond (ns) of production, or post-equilibrium, simulation. Each simulation was carried out using the isothermal-isobaric (NPT) ensemble (P = 1 atm, T = 300 K). During the production simulation, the DNA structures were recorded every picosecond for a total of 1000 frames of DNA structures. For each 7-mer and 8-mer, these 1000 frames were aligned to calculate the average DNA structure. From the average structure, we performed normal mode analysis [37] using the 3DNA software tool [34] to estimate six base parameters for the middle base pairs of 3-mers, and six step parameters for the middle base pairs of 4-mers. The six base parameters are shear, buckle, stretch, propeller, stagger and opening, and the six step parameters are shift, tilt, slide, roll, rise and twist [37].

#### Chemical features

A TF can recognize specific DNA sequences based on direct contact between nucleotides and amino acids through electrostatic and hydrophobic interactions. These molecular interactions, and therefore the interaction field features of a nucleotide, depend on nearby bases. Considering nucleotides beyond the first nearest neighbor bases did not result in significantly different values for interaction field features (results not shown), but it was significantly more computationally expensive. Thus, we considered only the influence of immediately adjacent bases in calculations of the molecular interaction field features of a nucleotide.

**Figure 1:**
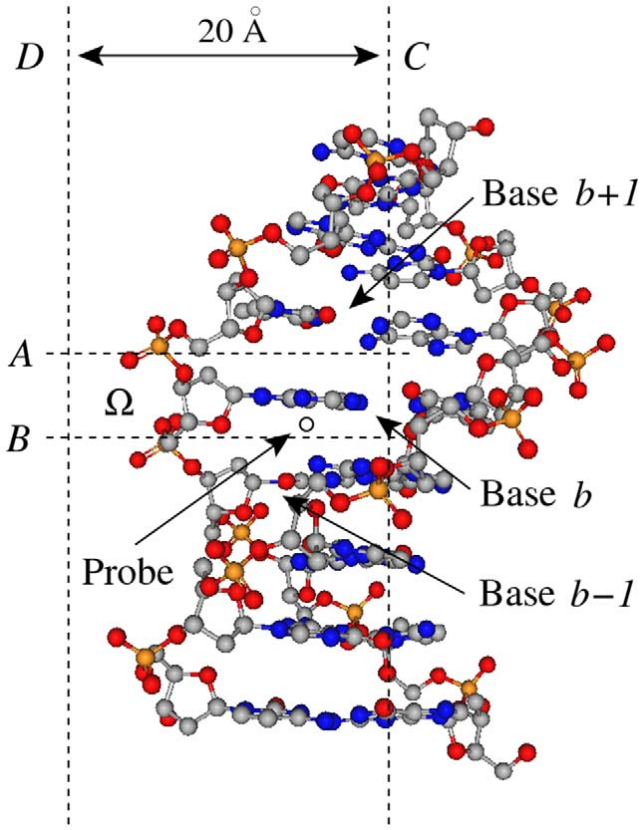
Computation of the molecular interaction potential. The local coordinate references for base pairs associated with bases *b*, *b+1*, and *b−1* are defined using the reference framework for the description of nucleic acid base-pair geometry [37]. The volume Ω is defined as the space constrained by four planes *A*, *B*, *C* and *D*. Plane *A* (*B*) bisects Bases *b* and *b+1* (*b* and *b−1*), and Plane *C* is perpendicular to Planes *A* and *B* and bisects Base *b* and its complementary base. Plane *D* marks a boundary 20 Å away from Plane *C*. Outside this area, the interaction energy tends to be weak (greater than −0.001 Kcal/mol). A probe is placed in Ω and the interaction energy between the DNA and the probe is calculated using the GRID software tool [38]. A total of 31 probes, listed in Table S1, are used in these calculations. See the Methods section for more details.

Let *b* be a middle nucleotide of a 3-mer as shown in Figure 1. To characterize the sequence-dependent molecular interaction field around *b*, we used the average structure for the 3-mer obtained from MD simulations and defined *Ω* as the volume around the base *b* constrained by four planes (*A*, *B*, *C*, and *D*) as shown in Figure 1. Within *Ω*, we systematically placed a small probe at different locations and computed the interaction energy between the DNA and the probe using the molecular force field encoded in GRID [38], a software tool designed for this purpose. We considered 31 probes available in GRID, such as an alkyl hydroxyl group, a methyl group, and an aliphatic neutral amide group (Table S1). The distance between planes *C* and *D*, which bound Ω, is 20 Å. This distance was chosen to capture all interactions between a probe and the DNA sequence that produce energy less than −0.001 Kcal/mol, which is the largest negative energy reported by GRID.

**Figure 2:**
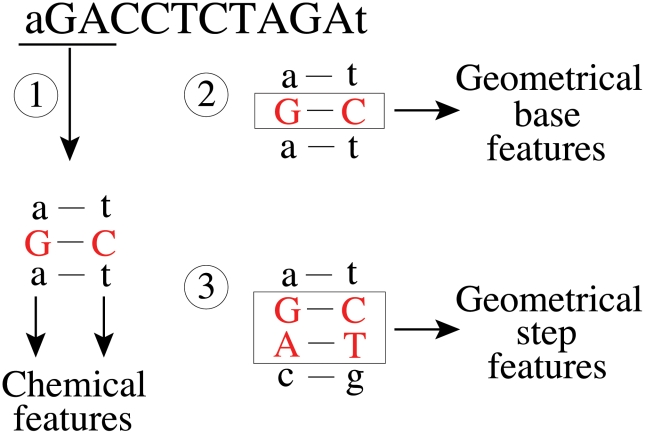
Mapping of DNA sequences to feature vectors. DNA sequences of known or potential TF binding sites are mapped to feature vectors as illustrated here for the 10-base sequence GACCTCTAGA. Red letters indicate nucleotides that are mapped to structural and chemical features and boxes indicate base pairs mapped to structural features. Step 1: map each of the ten nucleotides and its complement to eight chemical features. Step 2: map each middle base pair in the ten possible 3-mers to six geometrical base features. Step 3: for each of the nine possible 4-mers, map the two middle base pairs to six geometrical step features. For this example sequence, there are ten triplets and nine quadruplets, which result in a total of *n* = 274 feature vector components. A detailed description of the process of mapping DNA sequences to features is provided in the Methods section. The features associated with AGA are listed in Table S2.

For each probe *i* ∈ {1,…, 31}, using the GRID software tool [38], we calculated and recorded the minimum interaction energy, 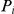:
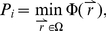
(2a)where 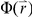 is the potential at point 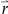. We also calculated the interaction score, 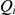:
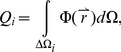
(2b)where the integration is performed over the volume Ω. We integrated over all points in Ω where the interaction energy was less than −0.001. The interaction field features for all middle bases in all of the 64 possible 3-mers were calculated and stored for use in defining chemical features as described below (Figures 1 and 2). For probe *i*, the interaction score, 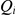, is a measure of the energy stored in the field of the DNA sequence in the volume Ω. Note that we defined the volume for each nucleotide separately rather than for a base pair to capture more information about DNA structure, such as major groove and minor groove effects.

A middle base of a 3-mer is associated with 62 molecular interaction field features: a minimum interaction energy given by Eq. 2a for each of the 31 probes and an interaction score given by Eq. 2b for each of the 31 probes. We found that some of these features are correlated. To identify a smaller set of uncorrelated features, we used principal component analysis (PCA). PCA generates a list of uncorrelated variables, or principal components, that are described by the eigenvectors of the correlation matrix of a dataset. The variability in the dataset is captured by the eigenvalues that correspond to the eigenvectors. For each probe and each of the 64 possible 3-mers, the values of the 62 molecular interaction field features for each base in the middle base pair were normalized to mean 0 and standard deviation 1 and organized in a 64×62 matrix. PCA was performed on this matrix. We arbitrarily chose the first eight eigenvalues, which capture 93% of the variance, and used the eigenvectors associated with these first eight eigenvalues as the chemical features to be used in training. Thus, for each middle base in a 3-mer, its chemical features are the corresponding elements from the first eight principal components, or eigenvectors, from PCA of the molecular interaction field features.

#### Mapping of DNA sequences to feature vectors

For a given TF that recognizes binding sites of length *L* in a genome, DNA sequences of length *L* are mapped to feature vectors as follows. For each of the *L* bases in a DNA sequence, we determine six geometrical base parameters and eight chemical features. These features are those that were calculated as described above for a 3-mer with the base of interest at the middle position. Recall that the eight chemical features are derived from the principal components of 62 molecular interaction field features. We also determine six geometrical step parameters for the middle two bases of all possible 4-mers. For efficiency, the features of a sequence are determined by table look up. In other words, the features of all possible 3- and 4-mers were calculated before assigning features to known and potential TF binding sites and saved in a table. Recall that structural features of 3- and 4-mers were determined in the context of flanking GC sequences.

Figure 2 illustrates how feature vectors are obtained for a particular DNA sequence. The features associated with a sequence depend on the flanking nucleotides. As shown in Step 1 of Figure 2, for each of the ten nucleotides in the DNA sequence GACCTCTAGA, starting with G, we determined the chemical features of the 3-mer in which this nucleotide is centered. Since DNA is double stranded, both strands were mapped to chemical features. For example, G within AGA and its complement taken in reverse, C within TCT, were mapped to chemical features. Then, shifting one base to the right, the next triplet GAC and its complement GTC were mapped to chemical features. This process continues until the last base in the sequence, A, is reached. The ten possible 3-mers for this example are AGA, GAC, ACC, CCT, CTC, TCT, CTA, TAG, AGA, and GAT. The corresponding reverse complements are TCT, GTC, GGT, AGG, GAG, AGA, TAG, CTA, TCT, and ATC. In Step 2 of Figure 2, we mapped each middle base pair in the ten possible 3-mers in the sequence to six geometrical base features. Similarly, in Step 3, we mapped the two middle base pairs for each of the nine possible 4-mers in the sequence to six geometrical step features, starting with GA in AGAC. The nine possible 4-mers for this example are AGAC, GACC, ACCT, CCTC, CTCT, TCTA, CTAG, TAGA, and AGAT. For this example sequence, there are ten triplets and nine quadruplets, which result in (10 triplets*8 features from PCA analysis per base*2 middle bases per triplet) 160 chemical features, (10 triplets*6 structural base features per triplet) 60 structural base features, and (9 quadruplets*6 structural step features per quadruplet) 54 structural step features, for a total of 274 feature vector components (*n* = 274). The structural and chemical features associated with AGA are given in Table S2 for reference.

#### Sources of negative and positive examples for training

The *E. coli* genome was downloaded from KEGG [39]. The *E. coli* open reading frames (ORFs) were identified in KEGG. For each *E. coli* TF, its documented binding sites were downloaded from RegulonDB 5.6. We decided to consider only *E. coli* TFs with at least five known binding sites. There are 54 such TFs in RegulonDB. The DNA sequences for the set of known binding sites for a given TF were mapped to feature vectors, and these vectors were used in training. To obtain negative examples for training, we first removed the ORFs from the genome. The remaining non-coding portions of the genome were taken to be negative examples of TF binding sites. We randomly selected 10,000 non-coding sequences to serve as negative examples for each TF, and mapped these sequences to feature vectors. We also obtained positive training data from DPInteract [40]. The source of training data did not affect the main qualitative findings of our method comparisons reported in the Results section. Namely, we find that the performance of SiteSleuth is better than the other methods tested. Results based on DPInteract training data are given in Table S5 of the Supplemental Material. These results are not discussed further because DPInteract has not been updated for some time and more binding sites are documented in RegulonDB.

To build a SiteSleuth model for a TF, we need known binding sites for the TF (positive examples), 10,000 randomly selected non-coding sequences (negative examples), and the structural and chemical features of short DNA sequences. It is time consuming to generate the structural and chemical features of short DNA sequences because these features require MD simulations to be performed and molecular interaction energy calculations. However, the MD simulations are performed only once and the structural and chemical features of short DNA sequences are tabulated. SiteSleuth classifiers are defined by a vector (**w** ^T^, d), whose determination requires SVM training by solving the minimization problem defined in Eq. 1a subject to the constraints defined in Eq. 1b for the positive and negative examples. We used libsvm [27] for training. A single training run takes less than 1 minute. For a potential binding site *m*, we used the tabulated structural and chemical features to calculate feature vector **x** *_m_* and the prediction value **w** ^T^ **x** *_m_*+d. Once this is done, using the SiteSleuth model to scan the *E.coli* genome requires several minutes for each TF.

### Implementation of other TF binding site prediction methods

For comparison, we implemented four other computational TF binding site prediction methods: the method of Berg and von Hippel (BvH) [3], Match [7], MATRIX SEARCH [5], and QPMEME [6]. These methods were implemented as described in the cited papers and, for the 54 TFs studied, a list of binding sites predicted by each method can be found online at http://cellsignaling.lanl.gov/EcoliTFs/SiteSleuth/. For completeness, each method is briefly presented below.

To discuss these methods we will need to first introduce a few quantities. For a set of *N* DNA binding sites of a particular TF, the length of each binding site is denoted by *L*. The value of *L* is set equal to the length of binding sites reported in RegulonDB for a given TF. In the case of Fis, we set *L* = 21. We define 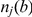 to be the number of times base *b* appears in the *j* ^th^ position in the sequences of the binding sites, and 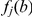 to be the corresponding frequency. We denote 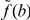 as the overall background frequency of base *b*. We use *S* to denote a potential TF binding site of length *L* and we use *S_j_* (*j* = 1,…, *L*) to denote the *j* ^th^ base of sequence *S*.

For the BvH method, we denoted the number of occurrences of the most common base in position *j* of the set of binding sites by 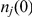. Using a training set of *N* binding sites, the BvH method calculates the score of each binding site as the summation over every position of the log-odds score of observing a base of *S* versus the most frequent base in the corresponding position of the sequence. Thus, the score is given by
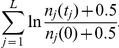
A pseudocount of 0.5 is used in the formula [3]. A cutoff threshold is defined as the mean score of the *N* positive training examples. To evaluate whether a new sequence *S* is a binding site, the score of *S* is calculated based on the above formula and compared with the cutoff threshold. If the score of sequence *S* is greater than the cutoff threshold, it is predicted to be a binding site.

For the Match method, a set of *N* training examples is used to define an information vector 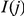, which describes the conservation of the position *j* in a binding site from the training set:
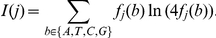
The information vector is used to evaluate whether a new sequence *S* is a binding site or not by calculating a score defined as
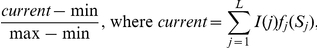
and min and max are calculated using the lowest and highest nucleotide frequency in each position, respectively. A cutoff threshold is defined as the mean score of the *N* positive training examples. If the score for a new sequence *S* is larger than the cutoff threshold, *S* is predicted to be a binding site.

Using a set of *N* binding sites as training examples, the MATRIX SEARCH method calculates the score of each binding site *S* as the summation over every position of the log-odds score of observing a base in *S* versus the overall background frequency of that base in the corresponding position of the sequences. Thus, the score is given by
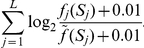
A pseudocount of 0.01 is used in the formula [5]. A cutoff threshold is determined as the mean of the *N* scores calculated from the training data. A new sequence *S* is predicted to be a binding site if its score is greater than the cutoff threshold.

The QPMEME (Quadratic Programming Method of Energy Matrix Estimation) method defines a weight 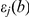 for each base *b* at position *j* in *S*. The score for a sequence *S* is defined as
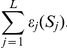
The weight 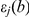 is estimated via a learning algorithm that only uses positive examples. The learning algorithm minimizes the variance 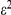 subject to the constraint that the score for each known binding site is less than a predefined cutoff value. Consistent with the Methods section of Djordjevic *et al.* [6], we used −1 for the cutoff value in our implementation of QPMEME, which constrains all known binding sites to one side of a hyperplane. Mathematically, the learning algorithm is described by
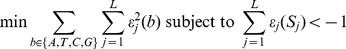
for every *S* in the training data set.

### Comparison of methods

#### Cross-validation

SiteSleuth was implemented for 54 TFs, which each have at least five known binding sites in *E. coli* according to RegulonDB (Table S3). A complete list of binding sites predicted by SiteSleuth for each TF can be found online at http://cellsignaling.lanl.gov/EcoliTFs/SiteSleuth/. A linear SVM served as the classification model for each TF. The classification models were used to scan the entire non-coding portion of the DNA sequence to predict new binding sites. For BvH, Match, and MATRIX SEARCH, as described above, the cutoff thresholds for classifying potential binding sites as true binding sites were defined to be the mean scores of the positive training examples. The cutoff threshold used for QPMEME was −1 [6]. The cutoff threshold for SiteSleuth was **w** ^T^ **x**+d>0.

Each model relies on a set of parameters, some of which are fixed and some of which are free parameters that must be estimated. More complex models have more free parameters, but these free parameters increase the chance of overfitting the data. It is possible that complex models will be able to fit the training data well but that the model's ability to accurately predict new TF binding sites may be low. Thus, to address the question of possible overfitting and to evaluate each model's prediction capability we performed 3-fold cross-validation. For each TF, training and testing were performed ten times to estimate the mean cross-validation value for the positive examples. The cross-validation score, *V*, is the fraction of positive examples predicted to be true binding sites.

One measure used to compare classifiers is the area under a receiver operating characteristics (ROC) curve. A ROC curve is a two-dimensional plot of the false positive rate (1 - specificity) versus the true positive rate (sensitivity). Each data point on this plot is generated by changing the cutoff values of classifiers and the area under the ROC curve (AUC) is calculated. The AUC is always between 0 and 1. A perfect classifier will have an AUC of 1 and a random classifier will have an AUC of 0.5. We implemented an algorithm for generating ROC curves and for calculating the AUC, which ranks classifier scores according to testing examples [41]. Positive examples for a given TF are chosen by randomly dividing the training set data into 2/3 positive training examples and 1/3 positive testing examples. The non-coding portions of the *E. coli* genome were used to generate all possible negative examples of TF binding sites. We built models using the training examples for the five methods. The models are used to calculate scores for positive testing examples and negative examples. An ROC curve and the corresponding AUC were estimated. For each TF, we performed the above procedure ten times to estimate ten AUCs for each method, and we reported the average value and standard deviation of AUC. For *n* positive testing examples, we can generate *n* points to draw the ROC curve. Fewer positive testing examples may generate large uncertainty in AUC calculation. Thus, we performed AUC analysis only for TFs in RegulonDB with at least 20 known binding sites.

#### Comparison with experimental data

We further interrogated the performance of these methods against SiteSleuth by comparing predictions against experimental data for Fis binding to *E. coli* DNA [17]. Cho *et al.* [17] identified 894 Fis-associated binding regions in ChIP-chip experiments. For each computational method, its list of predicted Fis binding sites, 21 base pairs (bp) in length, was compared to these 894 binding regions. Comparisons were made by scanning the binding region in the forward and reverse directions. A match was recorded if the complete predicted binding site or its complement was found within the experimentally determined binding region. False positives were computed by subtracting the number of matches from the total number of predicted binding sites.

## Results

### Local structural features of DNA depend on nucleotide environment

**Figure 3:**
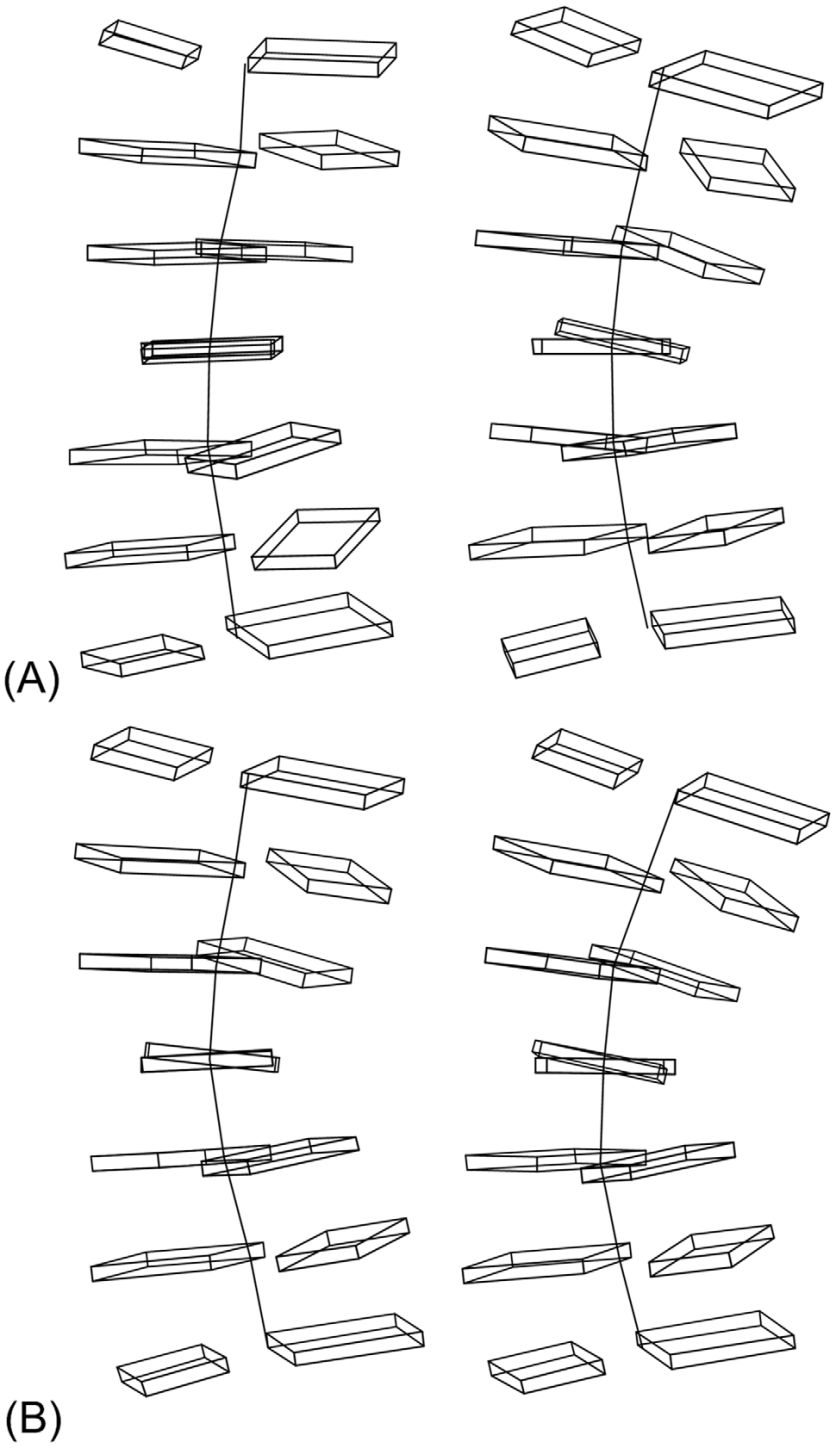
Structural features depend on nucleotide environment. These figures show the twist angle between the two base planes of a base pair in the vertical center of each of four DNA structures corresponding to the DNA sequences indicated below. All structures were obtained through MD simulations, as described in the Methods section. (**A**) Sequences with the same central base can have different properties in different local environments: **G** in GCT**G**GGC (left) is twisted −4.3 degrees relative to its cognate base and **G** in GCA**G**AGC (right) is twisted −20.4 degrees. (**B**) Sequences with different central bases can have similar structural properties: **A** in GCC**A**GGC (left) is twisted −9.5 degrees relative to its cognate base and **G** in GCC**G**GGC (right) is twisted −9.5 degrees.

To make a preliminary assessment of our hypothesis that we can produce better predictions if we consider the chemical and structural features of sequence-specific DNA, we examined the features of various sequences and found that the same base in the same position in a sequence can have different chemical and structural features depending on its environment. We illustrate this finding in Figure 3, which shows sequence-specific DNA structures. From the structures, one can see the context-dependent variation in the twist angle between the center two base planes. The center base pair is the same in each structure, but the twist angle for the left structure of Figure 3A is −20.4°, whereas the twist angle for the right structure of Figure 3A is −4.3°. Figure 3A demonstrates that different local structural features may characterize the same nucleotide at the same position in a sequence. The feature vectors for TGG and AGA are given in Table S2. Similarly, Figure 3B demonstrates that different nucleotides in the same position may be characterized by the same local structural features. The twist angles of the middle base pairs of the two structures in Figure 3B are the same, even though the base pairs are different. These observations suggested to us that chemical and structural features may capture sequence correlations relevant for TF-DNA interactions that are not apparent from sequence data alone and encouraged us to build classifiers that separate negative and positive examples of TF binding sites based on their positions in chemical and structural feature space. This approach, which we call the SiteSleuth method, combines DNA structure prediction, computational chemistry and machine learning.

To demonstrate the reliability of MD simulations for prediction of structural features of DNA oligomers, we calculated the propeller feature using 1) available experimental structural data (obtained from the Nucleic Acid Database [33]) and 2) predicted structures obtained via MD simulations, and we found significant correlation (about 0.8). The results are shown in Figure S2.

### Classifiers

As described in the Methods section, binary SiteSleuth classifiers were developed to identify and predict the binding sites of 54 TFs based on TF binding sites documented in RegulonDB. The input to a classifier is a vector of structural and chemical features generated from DNA sequences, each labeled as either a positive or negative example. Negative examples were taken from randomly chosen non-coding sequences of the *E. coli* genome. The classifiers were then used to scan both strands of non-coding sequences in the *E. coli* genome from 5′ to 3′ to identify potential TF binding sites. For comparison, we also considered four other computational TF binding site prediction methods: BvH [3], MATRIX SEARCH [5], Match [7], and QPMEME [6] These methods are each briefly described in the Methods section.

### Cross-validation of classifiers

**Figure 4:**
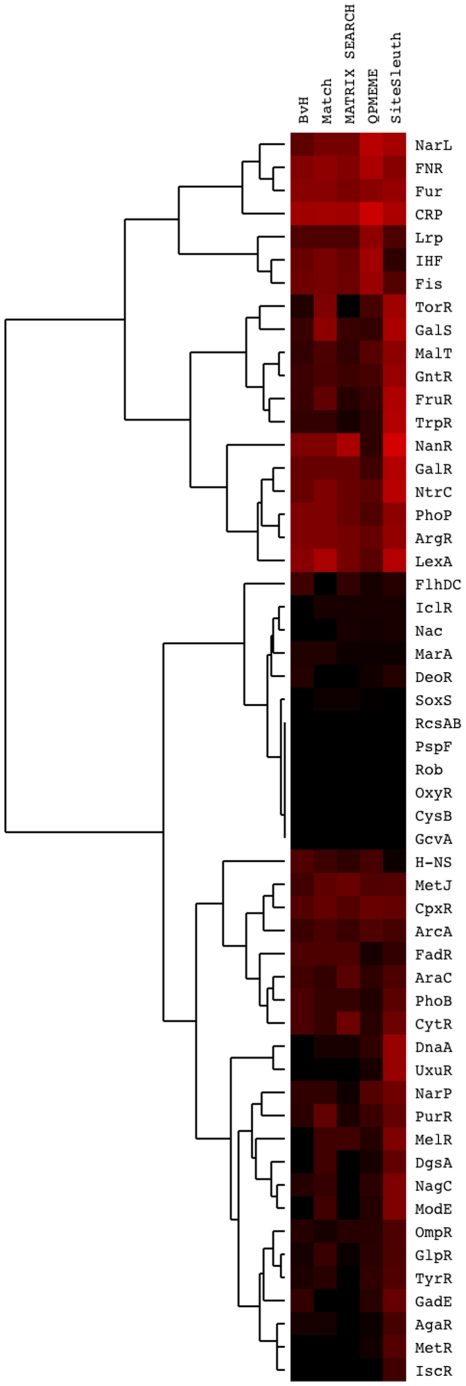
Cross-validation heat map. Heat map of cross-validation score, *V*, for the five methods indicated along the top for each of the 54 TFs indicated on the right. Bright red indicates a high cross-validation score, whereas black indicates *V = 0* (the lowest score). The highest score is *V = 1*. Of the 54 TFs studied, SiteSleuth outperforms all the other methods in 28 cases, equals the next best method in 11 cases, and performs more poorly in 15 cases. The ranking of methods in order of the number of times a method outperforms all the others is as follows: SiteSleuth (28)>QPMEME (8)>MATRIX SEARCH (2) = BvH (2)>Match (0).

The accuracy of predictions of each method was evaluated through a 3-fold cross-validation procedure, described in the Methods section. For each method, the mean cross-validation score, *V*, for the 54 TFs considered are listed in Table S4 and classifier accuracy is summarized in Figure 4. Recall that *V* is the fraction of positive examples predicted to be true binding sites in the cross-validation procedure.

Figure 4 is a heat map showing the cross-validation score, 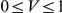, produced by each of the five computational methods. Brighter red indicates a higher cross-validation score and black represents 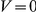. A cross-validation score of 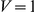 indicates perfect prediction, whereas a cross-validation score of zero indicates that the method fails to predict any TF binding sites correctly. Of the 54 TFs studied, SiteSleuth outperforms all the other methods in 28 cases, equals the next best method in 11 cases, and performs more poorly in 15 cases. Based on the number of times a method outperformed all the other methods, SiteSleuth (28) performed better than QPMEME (8), which performed better than MATRIX SEARCH (2), which equaled the performance of BvH (2), which performed better than Match (0). In one case, IcsR, SiteSleuth is the only method for which 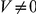. The data used to construct Figure 4 are given in Table S4.

Interestingly, Figure 4 reveals that all methods give cross-validation scores of zero for several TFs: CysB, GcvA, OxyR, RcsAB, and Rob. This observation suggests that methods that rely on DNA sequence information, including SiteSleuth, are insufficiently equipped to predict the binding sites for these TFs. Some of these TFs, such as GcvA [42], may perhaps recognize DNA indirectly via interaction with a second protein that recognizes DNA directly. Another explanation could be that some of these TFs, such as Rob [43], may be recognizing very short sequences.

**Figure 5:**
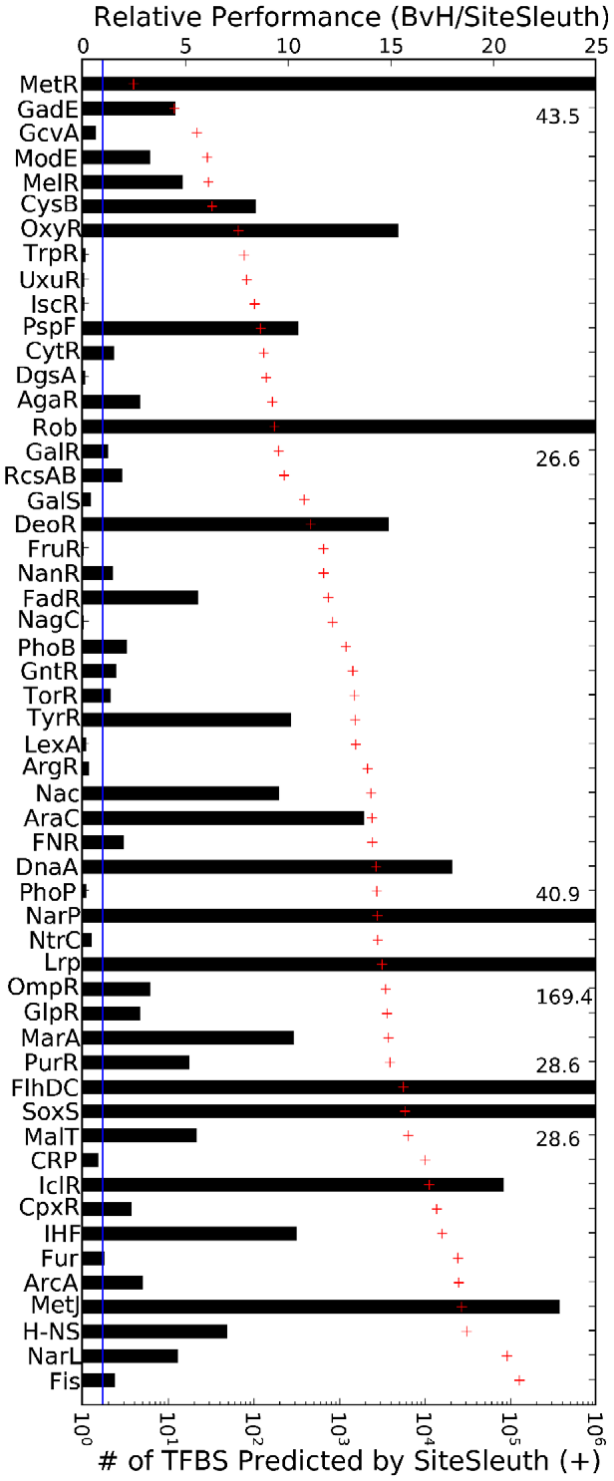
Bars show the relative performance (RP) of SiteSleuth compared to BvH. The quantity RP is defined as the number of predictions given by BvH divided by the number of predictions given by SiteSleuth. The value of RP is given on the top axis. A solid line is drawn at RP = 1. RP>1 indicates that BvH predicts a greater number of TF binding sites than SiteSleuth. The number of TF binding sites predicted by SiteSleuth (+) is indicated on the bottom axis. Of the 54 TFs tested, 13 TFs have RP<1 and 41 have RP>1. Taken together with the Fis ChIP-chip data [17], this figure shows that BvH predicts more estimated false positives than SiteSleuth. See the main text for further discussion.

The total number of TF binding sites predicted by each computational method is given in Table S3. For most TFs, QPMEME and Match both predict a large number of TF binding sites in the *E. coli* genome. The BvH and MATRIX SEARCH methods predict fewer binding sites, but still more than the number of predictions generated by SiteSleuth. In Figure 5, we show the performance of SiteSleuth relative to that of BvH for the TFs with five or more known binding sites. The relative performance (RP) score shown in Figure 5 is defined as the number of TF binding sites predicted by BvH divided by the number of TF binding sites predicted by SiteSleuth. This score indicates how many times more TF binding sites are predicted by BvH than by SiteSleuth. For example, BvH predicts 23 times more TF binding sites for MetJ than does SiteSleuth. For reference, the log transformed number of TF binding sites predicted by SiteSleuth is also indicated in Figure 5 and a solid line is drawn at RP = 1. As can be seen in Figure 5, 41 TFs have RP>1 and 13 TFs have RP<1. Thus, there is a large class of TFs for which SiteSleuth predicts fewer binding sites than BvH (RP>1) and, by extension, the other computational methods. From these results alone, it is not clear whether fewer predictions are a result of fewer false positives or more false negatives. To examine this question, we considered ChIP-chip data for Fis binding to DNA [17], which, as shown in Figure 5, has RP>1. Our findings are discussed in the next section.

As described in the Methods section, we also generated ROC curves and calculated AUC to compare classifiers. For each of the five computational methods and for TFs in RegulonDB with 20 or more known binding sites, the AUC values are tabulated in Table S6. We find that SiteSleuth had the largest AUC for 60% of the TFs tested, BvH had the largest AUC for 25% of the TFs, MATRIX SEARCH had the largest AUC for 10% of the TFs tested, QPMEME had the largest AUC for 5% of the TFs tested, and Match had the largest AUC for 0% of the TFs tested.

### Validation against ChIP-chip data

ChIP-chip assays have identified 894 DNA sequences that bind Fis in *E. coli* [17], which we used to validate the Fis binding sites predicted by each method. Looking at SiteSleuth results for Fis, SiteSleuth predicted 129,150 binding sites for Fis from a positive training set of 133 binding sites published in RegulonDB (Table S3), the second largest training set available for the 54 TFs we studied. The relative performance of SiteSleuth for Fis binding site prediction is close to one for three of the other methods under consideration (RP_BvH_ = 1.56, RP_Match_ = 2.03, RP_MATRIX SEARCH_ = 1.55, and RP_QPMEME_ = 11.67). SiteSleuth's cross-validation score for Fis (*V* = 0.33) is low (Table S4). The availability of empirical data on Fis binding, including a larger number of known binding sites in RegulonDB for training, and the indirect recognition mechanisms of Fis binding to DNA [33] suggested that Fis may provide a good example to test whether SiteSleuth, which accounts for DNA structure, performs better than the other methods, despite its low cross-validation score.

**Figure 6:**
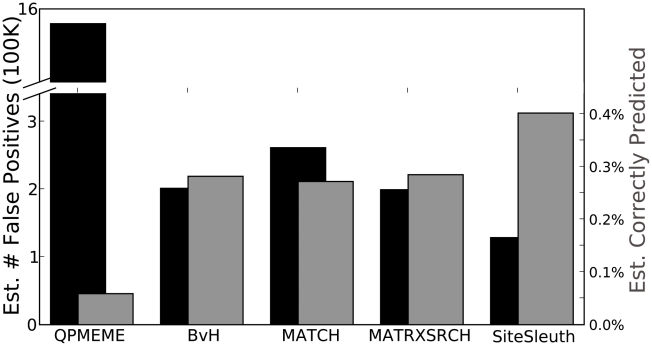
Evaluation of five computational methods using ChIP-chip characterization of Fis binding to *E. coli* DNA [**17**]. Black bars indicate the estimated number of false positives (left axis). Gray bars indicate the number of TF binding sites estimated to be correctly predicted divided by the total number of predictions (right axis). As described in the Methods section, the estimated number of false positives is calculated as the difference between a method's total number of predictions and the estimated number of Fis binding sites correctly predicted. SiteSleuth produces over 70,000 fewer false positives (difference between black bars for SiteSleuth and MATRIX SEARCH) and shows a 41% improvement in prediction accuracy over the next best method (compare the gray bars for MATRIX SEARCH and SiteSleuth).

Predictions of Fis binding sites from each computational method are compared to experimentally identified DNA sequences that bind Fis in *E. coli* in ChIP-chip assays [17]. We assume that the sequences found in this study contain, to a first approximation, the complete set of Fis binding sites. For each method, the approximate number of false positives was determined by subtracting the number of predictions that matched experimentally defined Fis binding sequences from the total number of predictions made by the method. Figure 6 shows the number of false positives generated by each computational method (black bars). As can be seen, the QPMEME method produced more than 1.5 million estimated false positives. Match generated approximately 261,000 false positives and BvH and MATRIX SEARCH both generated roughly 200,000 false positives. SiteSleuth produced the fewest false positives, over 70,000 fewer than the next best method, a reduction of 35% in the estimated false positive rate.

In absolute terms, QPMEME predicted a binding site within 889 of the 894 experimentally defined Fis binding sequences (99.44%). However, the predictions are not practically useful, since they are hidden within over 1.5 million estimated false positive results. The gray bars in Figure 6 report the percentage of TF binding sites correctly predicted by the five computational methods normalized by the total number of predictions. After normalization, QPMEME was the lowest performer for Fis. The BvH, Match, and MATRIX SEARCH methods gave approximately equivalent results. SiteSleuth outperformed these methods, showing a 41% improvement over MATRIX SEARCH, the next best method.

## Discussion

We postulated that a better TF binding site prediction method could be developed on the basis of chemical and structural features, instead of letter sequences. To test this hypothesis, we developed the SiteSleuth method, in which potential TF binding sites are associated with DNA sequence-specific structural and chemical features. These features are then used to build classification models for and to predict TF binding sites. Compared to the other computational methods we tested, including the three methods that use a PWM representation of TF binding sites (BvH, Match, and MATRIX SEARCH), our method provides a higher cross-validation accuracy. For 72% of the TFs studied, SiteSleuth cross-validation accuracy is as high as or higher than any other method (Table S4). SiteSleuth also generates 35% fewer estimated false positive results (Figure 6), and gives more accurate predictions (41% improvement over the next best method) for TF binding sites (Figure 6). In addition, the four other methods considered here each rely on the additivity assumption, which states that each nucleotide in a DNA binding site contributes to binding affinity in an independent fashion. In the study of Benos et al. [44], the additivity assumption was tested. In general, the additivity assumption holds rather well as shown by ddG measurements of mutated DNA sites in several protein-DNA complexes [44]. However, it was shown that additivity is a poor assumption for some cases [44]. SiteSleuth does not rely on the additivity assumption, which may partially explain its better performance.

It must be noted that none of the methods for predicting TF binding sites considered here can be deemed reliable when used alone. In Figure 6, although SiteSleuth indeed produces the highest fraction of correct predictions, the fraction of correct predictions is still small at 0.4%. Nonetheless, SiteSleuth constitutes an advance over existing methods and the approach warrants further investigation. The chemical and structural features we have considered are crude and additional determinants of specificity and other biologically relevant features, such as amino acid side chain interaction energy with DNA, could be incorporated into the SiteSleuth approach in the future. It may also be possible to incorporate experimental measurements of short DNA sequence properties into the SiteSleuth framework. A mechanistic understanding of TF binding to DNA could guide the design of novel model features. For example, a recent study of Fis showed that the shape of the DNA minor groove affects Fis-DNA binding [45]. This property is hard to capture using only DNA letter sequences, but could be captured by defining a new feature in SiteSleuth based on the available structural data. Presently, the features defined in SiteSleuth are unable to capture the effects of the minor groove on Fis binding, which may account for SiteSleuth's poor performance in absolute terms.

The QPMEME method is similar to the SVM-based approach of SiteSleuth. Both methods involve a quadratic programming minimization procedure with linear inequality constraints. QPMEME maps sequences of *L* bases into 4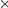 *L* multidimensional spaces with energy terms for each dimension and constructs a hyperplane such that all positive examples are located on one side of the plane. This quadratic optimization procedure defines a separating hyperplane by minimizing the variance of energies in an energy matrix so as to minimize the number of random sequences lying on the side of the plane that contains the positive examples. In contrast, the separating hyperplane of an SVM divides true binding sites from nonbinding sites with maximum margin. The distinction between random sequences, considered in QPMEME, and negative examples, considered in SiteSleuth, is important because sequences do not appear with equal probability in the *E. coli* genome, as is shown in Figure S1. SiteSleuth used negative examples directly sampled from non-coding regions of the *E. coli* genome.

In the report of Djordjevic *et al.* [6], the QPMEME method is applied to non-ORF regions of the *E. coli* genome to predict binding sites for 34 TFs, including Fis. For Fis, Table 1 of Ref. [6] indicates that QPMEME predicts 255 Fis binding sites, compared to the 1.5 million found with QPMEME in our hands (Table S3). To ensure that our implementation was correct, we applied QPMEME using the same training data set used by Djordjevic *et al.* [6] from DPInteract and were able to reproduce their weight matrix [6]. For Fis, RegulonDB reports 133 binding sites, compared to only 19 reported Fis binding sites in DPInteract. This difference in the size of the training data set (19 versus 133 positive examples of Fis binding sites) may be responsible for the difference in number of predicted binding sites (255 vs. 1.5 million). As can be seen by comparing the common entries in Table 1 of Ref. [6] and in Table S3, Fis is not an isolated example of QPMEME predicting a larger number of TF binding sites when the number of positive training examples is larger. It is also the case for the TFs ArcA, ArgR, CRP, CytR, DnaA, FadR, FarR, Fnr, FruR, GalR, GlpR, H-NS, IHF, LexA, LRP, MetJ, NagC, NarL, OmpR, SoxS, and TyrR. The QPMEME method may perform poorly for TFs with relatively large numbers of known binding sites because QPMEME requires that all positive examples be located on one side of a hyperplane in the space spanned by an energy matrix [6] (see Methods section). Thus, known binding sites that are outliers in this space may potentially expand the range of sequences considered to be binding sites, such that recall is maximized at the expense of precision. We have not systematically investigated the reasons underlying our observation that QPMEME performs poorly for the TFs identified above when using positive training data from RegulonDB, as such an investigation was beyond the intended scope of our study.

In summary, how TFs selectively bind to DNA is one of the least understood aspects of TF-mediated regulation of gene expression. An ability to better predict TF binding sites from small training data sets may advance our understanding of TF-DNA binding, and may reveal important insights into TF binding specificity, regulation and coordination of gene expression, and ultimately into gene function. A long-standing problem has been how to identify new TF binding sites given known binding sites. The accuracy and usefulness of computational methods for genome-wide TF binding site prediction has been limited by the inability to validate, verify, and inform these methods. Only recently has technology matured to the point that we can assay for TF binding sites on a genome-wide scale. This capability should allow us to critically evaluate predictions from computational methods and to develop methods that are more predictive than those currently available. Toward this end, the work presented here provides a starting point for future investigations of how TF binding site prediction can be improved by considering the physical and chemical aspects of TF-DNA binding.

## Supporting Information

Figure S1 Bars indicate the frequencies of triplet sequences that appear in non-coding regions of the E. coli genome. As can be seen, the non-coding genome sequence is not random, i.e., the assumption that sequences appear with equal probability is invalid.
(3.93 MB TIF)

Figure S2 Correlation of propeller feature from simulation and experimental DNA structure. We downloaded all asymmetric units of nucleic acid-containing structures determined by X-ray crystallography from the Nucleic Acid Database (http://ndbserver.rutgers.edu/) [33]. From these structures, we extracted 1,867 3D DNA structures. For each DNA structure, we used the 3DNA software tool to calculate the average propeller feature for each of 64 possible middle bases of the 3-mers. The values of the propeller feature (x-axis) are plotted vs. the corresponding propeller features of average DNA structure from molecular dynamics simulation (y-axis). The correlation coefficient is 0.8, which shows good agreement.
(0.01 MB EPS)

Table S1 Probe types from GRID [38] used to estimate the molecular interaction field features Pi and Qi for probe type i as described in Fig. 1 and the Methods section. Definitions of the minimum interaction energy, Pi, and interaction score, Qi, are given in the Methods section (Eq. 2).
(0.14 MB DOC)

Table S2 Example showing different feature vectors given different nucleotide environments: G in TGG and G in AGA. Cross reference with Figs. 2 and 3.
(0.23 MB DOC)

Table S3 Computational results for 54 TFs, whose number of known binding sites documented in RegulonDB is five or more. For each computational method, the training set size and the number of predicted binding sites are given. See http://cellsignaling.lanl.gov/EcoliTFs/SiteSleuth/ for the complete listing of binding sites predicted by each method for each TF. The relative performance of BvH vs. SiteSleuth is plotted in Fig. 5 along with the log transformed number of predicted binding sites for SiteSleuth.
(0.25 MB DOC)

Table S4 For each method, mean cross-validation score (V), which is defined as the fraction of positive examples predicted to be true binding sites, for 54 TFs whose number of known binding sites documented in RegulonDB is five or more.
(0.26 MB DOC)

Table S5 Computational results for 44 TFs documented in DPInteract. Here, the cutoff values for the BvH, Match, and MATRIX SEARCH methods were each set to the lowest scoring sequence in the training set from which a model for a TF binding site was built. This approach, which guarantees that positive examples used in training are correctly classified, is different from that described in the Methods section. For the QPMEME method, cutoff values are set to −1, and for the SiteSlueth method, cutoff values are set to 0. For each method, the training set size and the number of predicted binding sites are given. In each case, the number of hits is approximately the same as that reported in [6]. The cross-validation score V is given in parentheses. In cross-validation, the available positive examples are divided into a training set and a testing set, as described in the main text. Models are built based on the training set and tested using the remaining positive examples. Recall that each model (derived through any of the five methods that we consider here) is built to ensure that the binding sites in the training set are classified correctly; however, the testing examples withheld from training may not be predicted perfectly by a method. Although the QPMEME method usually predicts a lower number of binding sites compared to any of the other methods, its cross-validation score is relatively low in most cases. These results are not discussed in the main text.
(0.18 MB DOC)

Table S6 Area under the curve (AUC) analysis for transcription factors (TFs) in RegulonDB with at least 20 known binding sites. A receiver operating characteristics (ROC) curve is a two-dimensional plot of the false positive rate (1 - specificity) versus the true positive rate (sensitivity). The AUC for an ROC curve is between 0 and 1. A perfect classifier will have an AUC of 1 and a random classifier will have an AUC of 0.5. We implemented an algorithm for generating ROC curves and for calculating AUCs, which allows us to rank classifiers. Positive examples in ReglonDB for a given TF are randomly divided into 2/3 positive training examples and 1/3 positive testing examples. The non-coding portions of the E. coli genome were used to generate all possible negative examples of TF binding sites. We built classifier models using the training examples for the five methods under consideration. The models are used to calculate scores for positive testing examples and negative examples. An ROC curve and the corresponding AUC were estimated from these scores for each model. For each TF, we performed the above procedure ten times to estimate ten AUCs for each method, and we report the average value and standard deviation of AUC in this table.
(0.07 MB DOC)

## References

[1] Wall ME, Hlavacek WS, Savageau MA (2003). Design Principles for Regulator Gene Expression in a Repressible Gene Circuit.. J Mol Biol.

[2] Lee SK, Keasling JD, Smolke CD (2010). Practical pathway engineering - demonstration in integrating tools.. The Metabolic Pathway Engineering Handbook: Tools and Applications.

[3] Berg O, von Hippel P (1987). Selection of DNA binding sites by regulatory proteins. Statistical-mechanical theory and application to operators and promoters.. J Mol Biol.

[4] Cartharius K, Frech K, Grote K, Klocke B, Haltmeier M (2005). MatInspector and beyond: promoter analysis based on transcription factor binding sites.. Bioinformatics.

[5] Chen QK, Hertz GZ, Stormo GD (1995). MATRIX SEARCH 1.0: a computer program that scans DNA sequences for transcriptional elements using a database of weight matrices.. Comput Appl Biosci.

[6] Djordjevic M, Sengupta AM, Shraiman BI (2003). A Biophysical Approach to Transcription Factor Binding Site Discovery.. Genome Res.

[7] Kel AE, Gössling E, Reuter I, Cheremushkin E, Kel-Margoulis OV (2003). MATCH™: a tool for searching transcription factor binding sites in DNA sequences.. Nucleic Acid Res.

[8] Osada R, Zaslavsky E, Singh M (2004). Comparative analysis of methods for representing and searching for transcription factor binding sites.. Bioinformatics.

[9] Quandt K, Frech K, Karas H, Wingender E, Werner T (1995). Matlnd and Matlnspector: new fast and versatile tools for detection of consensus matches in nucleotide sequence data.. Nucleic Acid Res.

[10] Stormo G (2000). DNA binding sites: representation and discovery.. Bioinformatics.

[11] Benos PV, Lapedes AS, Stormo GD (2002). Probabilistic Code for DNA Recognition by Proteins of the EGR Family.. J Mol Biol.

[12] Kaplan T, Friedman N, Margalit H (2005). Ab Initio Prediction of Transcription Factor Targets Using Structural Knowledge.. PLoS Comput Biol.

[13] Endres RG, Schulthess TC, Wingreen NS (2004). Toward an atomistic model for predicting transcription-factor binding sites.. Proteins.

[14] Morozov AV, Havranek JJ, Baker D, Siggia ED (2005). Protein-DNA binding specificity predictions with structural models.. Nucleic Acid Res.

[15] Morozov AV, Siggia ED (2007). Connecting protein structure with predictions of regulatory sites.. Proc Natl Acad Sci U S A.

[16] Berger MF, Bulyk ML (2009). Universal protein-binding microarrays for the comprehensive characterization of the DNA-binding specificities of transcription factors.. Nat Protoc.

[17] Cho B-K, Knight EM, Barrett CL, Palsson BO (2008). Genome-wide analysis of Fis binding in *Escherichia coli* indicates a causative role for A-/AT-tracts.. Genome Res.

[18] Maerkl SJ, Quake SR (2007). A Systems Approach to Measuring the Binding Energy Landscapes of Transcription Factors.. Science.

[19] Sarai A, Kono H (2005). Protein-DNA recognition patterns and predictions.. Annu Rev Biophys Biomol Struct.

[20] Becker NB, Wolff L, Everaers R (2006). Indirect readout: detection of optimized subsequences and calculation of relative binding affinities using different DNA elastic potentials.. Nucleic Acid Res.

[21] Zhang Y, Xi Z, Hegde RS, Shakked Z, Crothers DM (2004). Predicting indirect readout effects in protein-DNA interactions.. Proc Natl Acad Sci U S A.

[22] Ahmad S, Kono H, Arauzo-Bravo MJ, Sarai A (2006). ReadOut: structure-based calculation of direct and indirect readout energies and specificities for protein-DNA recognition.. Nucleic Acid Res.

[23] Gromiha M, Siebers JG, Selvaraj S, Kono H, Sarai A (2004). Intermolecular and Intramolecular Readout Mechanisms in Protein-DNA Recognition.. J Mol Biol.

[24] Gama-Castro S, Jimenez-Jacinto V, Peralta-Gil M, Santos-Zavaleta A, Penaloza-Spinola MI (2008). RegulonDB (version 6.0): gene regulation model of Escherichia coli K-12 beyond transcription, active (experimental) annotated promoters and Textpresso navigation.. Nucleic Acid Res.

[25] Aparicio O, Geisberg J, Struhl K (2004). Chromatin immunoprecipitation for determining the association of proteins with specific genomic sequences *in vivo*.. Curr Protoc Cell Biol.

[26] Vapnik V (1998). Statistical Learning Theory.

[27] Chang C-C, Lin C-J (2001). LIBSVM: a library for support vector machines, version 2.89.. http://www.csie.ntu.edu.tw/~cjlin/libsvm.

[28] Cheatham TE (2004). Simulation and modeling of nucleic acid structure, dynamics and interactions.. Curr Opin Struct Biol.

[29] Orozco M, Noy A, Pérez A (2008). Recent advances in the study of nucleic acid flexibility by molecular dynamics.. Curr Opin Struct Biol.

[30] Beveridge DL, Barreiro G, Suzie Byun K, Case DA, Cheatham TE (2004). Molecular Dynamics Simulations of the 136 Unique Tetranucleotide Sequences of DNA Oligonucleotides. I. Research Design and Results on d(CpG) Steps.. Biophys J.

[31] Dixit SB, Beveridge DL, Case DA, Cheatham TE, Giudice E (2005). Molecular Dynamics Simulations of the 136 Unique Tetranucleotide Sequences of DNA Oligonucleotides. II: Sequence Context Effects on the Dynamical Structures of the 10 Unique Dinucleotide Steps.. Biophys J.

[32] Lavery R, Zakrzewska K, Beveridge D, Bishop TC, Case DA (2010). A systematic molecular dynamics study of nearest-neighbor effects on base pair and base pair step conformations and fluctuations in B-DNA.. Nucleic Acid Res.

[33] Berman HM, Olson WK, Beveridge DL, Westbrook J, Gelbin A (1992). The nucleic acid database. A comprehensive relational database of three-dimensional structures of nucleic acids.. Biophys J.

[34] Lu X-J, Olson WK (2003). 3DNA: a software package for the analysis, rebuilding and visualization of three-dimensional nucleic acid structures.. Nucleic Acid Res.

[35] Phillips JC, Braun R, Wang W, Gumbart J, Tajkhorshid E (2005). Scalable molecular dynamics with NAMD.. J Comput Chem.

[36] Foloppe N, Alexander D, MacKerell J (2000). All-atom empirical force field for nucleic acids: I. Parameter optimization based on small molecule and condensed phase macromolecular target data.. J Comput Chem.

[37] Olson WK, Bansal M, Burley SK, Dickerson RE, Gerstein M (2001). A standard reference frame for the description of nucleic acid base-pair geometry.. J Mol Biol.

[38] Goodford PJ (1985). A computational procedure for determining energetically favorable binding sites on biologically important macromolecules.. J Med Chem.

[39] Kanehisa M, Goto S, Hattori M, Aoki-Kinoshita KF, Itoh M (2006). From genomics to chemical genomics: new developments in KEGG.. Nucleic Acids Res.

[40] Robison K, McGuire AM, Church GM (1998). A comprehensive library of DNA-binding site matrices for 55 proteins applied to the complete *Escherichia coli* K-12 genome.. J Mol Biol.

[41] Fawcett T (2006). An introduction to ROC analysis.. Pattern Recognition Letters.

[42] Heil G, Stauffer LT, Stauffer GV (2002). Glycine binds the transcriptional accessory protein GcvR to disrupt a GcvA/GcvR interaction and allow GcvA-mediated activation of the *Escherichia coli* gcvTHP operon.. Microbiology.

[43] Kwon HJ, Bennik MHJ, Demple B, Ellenberger T (2000). Crystal structure of the *Escherichia coli* Rob transcription factor in complex with DNA.. Nat Struct Mol Biol.

[44] Benos PV, Bulyk ML, Stormo GD (2002). Additivity in protein-DNA interactions: how good an approximation is it?. Nucleic Acid Res.

[45] Stella S, Cascio D, Johnson RC (2010). The shape of the DNA minor groove directs binding by the DNA-bending protein Fis.. Genes Dev.

