# Supplementary figures and images for: Using Sequence-Specific Chemical and Structural Properties of DNA to Predict Transcription Factor Binding Sites
Source: PLoS Comput Biol. 2010 Nov 18;6(11):e1001007. doi: 10.1371/journal.pcbi.1001007 (PMC2987836; doi:10.1371/journal.pcbi.1001007)

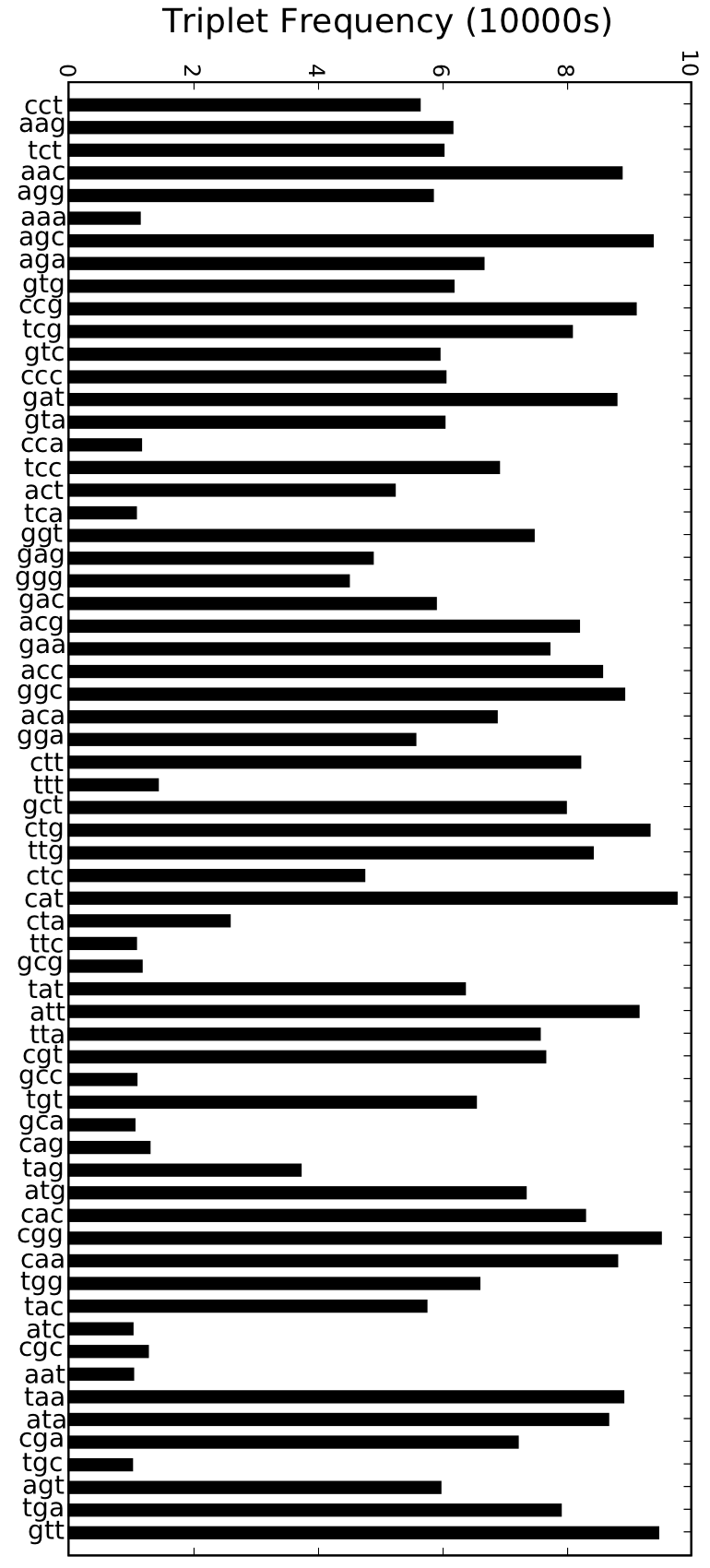

Supplement: Figure S1 — Bars indicate the frequencies of triplet sequences that appear in non-coding regions of the E. coli genome. As can be seen, the non-coding genome sequence is not random, i.e., the assumption that sequences appear with equal probability is invalid. (3.93 MB TIF) [file pcbi.1001007.s001.tif]
