# Supplementary material for: Using Sequence-Specific Chemical and Structural Properties of DNA to Predict Transcription Factor Binding Sites
Source: PLoS Comput Biol. 2010 Nov 18;6(11):e1001007. doi: 10.1371/journal.pcbi.1001007 (PMC2987836; doi:10.1371/journal.pcbi.1001007)
Supplement: Table S1 — Probe types from GRID [38] used to estimate the molecular interaction field features Pi and Qi for probe type i as described in Fig. 1 and the Methods section. Definitions of the minimum interaction energy, Pi, and interaction score, Qi, are given in the Methods section (Eq. 2). (0.14 MB DOC) [file pcbi.1001007.s003.doc]

**Table S1.** Probe types from GRID [38] used to estimate the molecular interaction field features *P_i_* and *Q_i_* for probe type *i* as described in Fig. 1 and the Methods section. Definitions of the minimum interaction energy, *P_i_*, and interaction score, *Q_i_*, are given in the Methods section (Eq. 2).

| Symbol | Probe Name in GRID |
| --- | --- |
| C3 | Methyl CH3 group |
| C1= | sp2 CH aromatic or vinyl |
| N:# | sp N with lone pair |
| N:= | sp2 N with lone pair |
| N: | sp3 N with lone pair |
| N-: | Anionic tetrazole N |
| N1 | Neutral flat NH eg amide |
| N1+ | sp3 amine NH cation |
| N1= | sp2 Amine NH cation |
| N1: | sp3 NH with lone pair |
| NH= | sp2 NH with lone pair |
| N1# | sp NH with one hydrogen |
| N2 | Neutral flat NH2 eg amide |
| N2+ | sp3 amine NH2 cation |
| N2= | sp2 Amine NH2 cation |
| N2: | sp3 NH2 with lone pair |
| N3+ | sp3 amine NH3 cation |
| NM3 | trimethyl-ammonium cation |
| O1 | Alkyl hydroxy OH group |
| OH | Phenol or carboxy OH |
| O- | sp2 phenolate oxygen |
| O | sp2 carbonyl oxygen |
| O:: | sp2 carboxy oxygen atom |
| O= | O of sulphate/sulphonamide |
| OES | sp3 ester oxygen atom |
| OC2 | Ether or furan oxygen |
| OS | O of sulphone / sulphoxide |
| ON | Oxygen of nitro group |
| OH2 | Water |
| BOTH | The Amphipatic Probe |
| DRY | The Hydrophobic Probe |
