# Supplementary material for: Using Sequence-Specific Chemical and Structural Properties of DNA to Predict Transcription Factor Binding Sites
Source: PLoS Comput Biol. 2010 Nov 18;6(11):e1001007. doi: 10.1371/journal.pcbi.1001007 (PMC2987836; doi:10.1371/journal.pcbi.1001007)
Supplement: Table S2 — Example showing different feature vectors given different nucleotide environments: G in TGG and G in AGA. Cross reference with Figs. 2 and 3. (0.23 MB DOC) [file pcbi.1001007.s004.doc]

**Table S2.** Example showing different feature vectors given different nucleotide environments: G in T**G**G and G in A**G**A. Cross reference with Figs. 2 and 3.

|  | **TGG** |  | **AGA** |  |
| --- | --- | --- | --- | --- |
| **Probes** | **P_i_** | **Q_i_** | **P_i_** | **Q_i_** |
| C3 | -3.248 | -128.201 | -2.942 | -150.578 |
| C1= | -3.082 | -129.678 | -2.703 | -153.563 |
| N:# | -7.604 | -313.089 | -7.723 | -360.015 |
| N:= | -6.815 | -230.811 | -6.824 | -258.561 |
| N: | -6.287 | -168.519 | -6.194 | -182.215 |
| N-: | -5.181 | -127.883 | -5.032 | -147.999 |
| N1 | -6.217 | -401.846 | -6.464 | -550.058 |
| N1+ | -17.043 | -593.572 | -16.051 | -755.213 |
| N1= | -17.62 | -630.153 | -16.589 | -799.727 |
| N1: | -8.606 | -443.91 | -8.24 | -588.04 |
| NH= | -7.411 | -444.305 | -7.034 | -586.5 |
| N1# | -14.829 | -642.447 | -13.305 | -822.858 |
| N2 | -8.024 | -436.57 | -7.746 | -593.011 |
| N2+ | -18.442 | -593.755 | -16.569 | -757.226 |
| N2= | -19.132 | -623.309 | -17.257 | -792.937 |
| N2: | -8.586 | -467.308 | -8.397 | -620.213 |
| N3+ | -16.737 | -566.788 | -16.867 | -714.089 |
| NM3 | -7.3 | -63.139 | -6.454 | -52.309 |
| O1 | -7.088 | -345.276 | -6.616 | -464.848 |
| OH | -6.838 | -356.244 | -6.379 | -482.177 |
| O- | -5.341 | -117.316 | -5.627 | -140.403 |
| O | -4.618 | -69.451 | -4.827 | -73.104 |
| O:: | -5.617 | -134.552 | -5.91 | -158.703 |
| O= | -4.432 | -141.821 | -4.551 | -172.264 |
| OES | -2.201 | -46.187 | -2.307 | -54.549 |
| OC2 | -3.384 | -54.823 | -3.533 | -58.362 |
| OS | -4.537 | -79.663 | -4.666 | -76.384 |
| ON | -4.893 | -236.881 | -4.968 | -278.126 |
| OH2 | -7.019 | -355.308 | -6.736 | -477.178 |
| BOTH | -0.522 | -0.544 | -0.372 | -0.108 |
| DRY | -0.93 | -7.573 | -0.886 | -1.798 |
| **Base Parameters** | | **TGG** | **AGA** |  |
| Shear |  | -0.23 | -0.22 |  |
| Stretch |  | -0.18 | -0.15 |  |
| Stagger |  | -0.16 | -0.02 |  |
| Buckle |  | 2.12 | 5.08 |  |
| Propeller |  | -4.34 | -20.39 |  |
| Opening |  | -1.1 | 0.86 |  |
| **Step Parameters** | | **TGGG** | **AGAG** |  |
| Shift |  | -0.05 | -0.38 |  |
| Slide |  | -0.25 | 0.29 |  |
| Rise |  | 3.44 | 3.24 |  |
| Tilt |  | -0.16 | -2.43 |  |
| Roll |  | 5.46 | 4.52 |  |
| Twist |  | 33.9 | 38.15 |  |
