# Supplementary material for: Using Sequence-Specific Chemical and Structural Properties of DNA to Predict Transcription Factor Binding Sites
Source: PLoS Comput Biol. 2010 Nov 18;6(11):e1001007. doi: 10.1371/journal.pcbi.1001007 (PMC2987836; doi:10.1371/journal.pcbi.1001007)
Supplement: Table S3 — Computational results for 54 TFs, whose number of known binding sites documented in RegulonDB is five or more. For each computational method, the training set size and the number of predicted binding sites are given. See http://cellsignaling.lanl.gov/EcoliTFs/SiteSleuth/ for the complete listing of binding sites predicted by each method for each TF. The relative performance of BvH vs. SiteSleuth is plotted in Fig. 5 along with the log transformed number of predicted binding sites for SiteSleuth. (0.25 MB DOC) [file pcbi.1001007.s005.doc]

**Table S3.** Computational results for 54 TFs whose number of known binding sites documented in RegulonDB is five or more. For each computational method, the training set size and the number of predicted binding sites are given. See <http://cellsignaling.lanl.gov/EcoliTFs/SiteSleuth/> for the complete listing of binding sites predicted by each method for each TF. The relative performance of BvH vs. SiteSleuth is plotted in Fig. 5 along with the log transformed number of predicted binding sites for SiteSleuth.

| **Name** | **Training Set Size** | **BvH** | **Match** | **MATRIX**  **SEARCH** | **QPMEME** | **Site**  **Sleuth** |
| --- | --- | --- | --- | --- | --- | --- |
| AgaR | 11 | 465 | 2236 | 360 | 1884 | 167 |
| AraC | 20 | 33412 | 52734 | 33390 | 102457 | 2442 |
| ArcA | 91 | 73895 | 76283 | 72860 | 132264 | 25229 |
| ArgR | 24 | 671 | 1686 | 652 | 18912 | 2171 |
| CpxR | 33 | 33101 | 55743 | 33221 | 368930 | 13964 |
| CRP | 260 | 7639 | 11030 | 7580 | 1069283 | 10189 |
| CysB | 8 | 278 | 1551 | 146 | 729 | 33 |
| CytR | 14 | 202 | 1505 | 175 | 2599 | 133 |
| DeoR | 7 | 6957 | 16652 | 6191 | 20923 | 467 |
| DgsA | 8 | 17 | 224 | 13 | 84 | 142 |
| DnaA | 10 | 48972 | 108426 | 47570 | 76486 | 2724 |
| FadR | 10 | 4238 | 14726 | 3920 | 33838 | 754 |
| Fis | 133 | 202096 | 262224 | 199966 | 1506632 | 129150 |
| FlhDC | 20 | 162411 | 234753 | 163533 | 492630 | 5688 |
| FNR | 85 | 4900 | 8543 | 4928 | 340882 | 2463 |
| FruR | 13 | 21 | 97 | 21 | 263 | 661 |
| Fur | 54 | 25843 | 37387 | 25838 | 275020 | 24684 |
| GadE | 5 | 54 | 368 | 29 | 255 | 12 |
| GalR | 10 | 244 | 996 | 206 | 3769 | 198 |
| GalS | 9 | 149 | 528 | 126 | 2591 | 395 |
| GcvA | 5 | 14 | 141 | 11 | 49 | 22 |
| GlpR | 23 | 10195 | 27198 | 10063 | 31268 | 3661 |
| GntR | 17 | 2358 | 7342 | 2317 | 23530 | 1455 |
| H-NS | 34 | 218778 | 303046 | 218934 | 597416 | 31154 |
| IclR | 10 | 232538 | 319893 | 235576 | 294673 | 11357 |
| IHF | 87 | 167137 | 316148 | 167122 | 1050684 | 16056 |
| IscR | 8 | 7 | 50 | 5 | 30 | 104 |
| LexA | 24 | 259 | 1745 | 254 | 30290 | 1578 |
| Lrp | 84 | 541474 | 625587 | 539556 | 1319479 | 3196 |
| MalT | 20 | 35743 | 55431 | 35261 | 125337 | 6468 |
| MarA | 16 | 39156 | 85183 | 38597 | 137897 | 3808 |
| MelR | 8 | 146 | 680 | 129 | 1114 | 30 |
| MetJ | 27 | 634694 | 1137063 | 621589 | 1449353 | 27334 |
| MetR | 6 | 174 | 800 | 114 | 778 | 4 |
| ModE | 8 | 95 | 765 | 73 | 724 | 29 |
| Nac | 10 | 22752 | 48020 | 21363 | 53086 | 2382 |
| NagC | 14 | 27 | 110 | 24 | 100 | 843 |
| NanR | 6 | 973 | 973 | 973 | 15678 | 664 |
| NarL | 91 | 426754 | 472550 | 431808 | 1682666 | 92451 |
| NarP | 16 | 115566 | 129839 | 115658 | 216367 | 2823 |
| NtrC | 22 | 1248 | 6264 | 1200 | 14721 | 2843 |
| OmpR | 20 | 11612 | 18694 | 11637 | 34207 | 3532 |
| OxyR | 9 | 1029 | 3870 | 768 | 2397 | 67 |
| PhoB | 14 | 2579 | 9954 | 2537 | 35604 | 1211 |
| PhoP | 22 | 470 | 1778 | 437 | 6042 | 2781 |
| PspF | 5 | 1280 | 4727 | 1116 | 2671 | 122 |
| PurR | 18 | 20483 | 81537 | 19980 | 118837 | 3956 |
| RcsAB | 5 | 442 | 1011 | 382 | 910 | 230 |
| Rob | 6 | 4712 | 17587 | 4019 | 9418 | 177 |
| SoxS | 18 | 170007 | 403160 | 168981 | 265797 | 5940 |
| TorR | 8 | 2060 | 6429 | 2027 | 9394 | 1520 |
| TrpR | 10 | 10 | 16 | 9 | 68 | 78 |
| TyrR | 19 | 15695 | 40645 | 15431 | 54411 | 1551 |
| UxuR | 5 | 5 | 7 | 5 | 8 | 84 |
