# Supplementary material for: Using Sequence-Specific Chemical and Structural Properties of DNA to Predict Transcription Factor Binding Sites
Source: PLoS Comput Biol. 2010 Nov 18;6(11):e1001007. doi: 10.1371/journal.pcbi.1001007 (PMC2987836; doi:10.1371/journal.pcbi.1001007)
Supplement: Table S4 — For each method, mean cross-validation score (V), which is defined as the fraction of positive examples predicted to be true binding sites, for 54 TFs whose number of known binding sites documented in RegulonDB is five or more. (0.26 MB DOC) [file pcbi.1001007.s006.doc]

**Table S4.** For each method, mean cross-validation score (*V*), which is defined as the fraction of positive examples predicted to be true binding sites, for 54 TFs whose number of known binding sites documented in RegulonDB is five or more.

| **TF** |  |  | **MATRIX** |  |  |
| --- | --- | --- | --- | --- | --- |
| **Name** | **BvH** | **Match** | **SEARCH** | **QPMEME** | **SiteSleuth** |
| AgaR | 0.09 | 0.09 | 0 | 0.05 | 0.27 |
| AraC | 0.25 | 0.20 | 0.35 | 0.18 | 0.30 |
| ArcA | 0.23 | 0.29 | 0.23 | 0.31 | 0.27 |
| ArgR | 0.50 | 0.50 | 0.46 | 0.40 | 0.58 |
| CpxR | 0.33 | 0.39 | 0.33 | 0.41 | 0.39 |
| CRP | 0.63 | 0.64 | 0.63 | 0.80 | 0.67 |
| CysB | 0 | 0 | 0 | 0 | 0 |
| CytR | 0.29 | 0.21 | 0.43 | 0.16 | 0.43 |
| DeoR | 0.14 | 0 | 0 | 0.07 | 0.14 |
| DgsA | 0 | 0.25 | 0 | 0.10 | 0.38 |
| DnaA | 0 | 0.10 | 0.10 | 0.19 | 0.60 |
| FadR | 0.30 | 0.30 | 0.30 | 0.10 | 0.20 |
| Fis | 0.44 | 0.47 | 0.43 | 0.61 | 0.33 |
| FlhDC | 0.25 | 0 | 0.20 | 0.09 | 0.15 |
| FNR | 0.51 | 0.56 | 0.51 | 0.67 | 0.53 |
| FruR | 0.23 | 0.38 | 0.15 | 0.22 | 0.69 |
| Fur | 0.54 | 0.54 | 0.48 | 0.54 | 0.59 |
| GadE | 0.20 | 0 | 0 | 0.16 | 0.40 |
| GalR | 0.40 | 0.40 | 0.40 | 0.25 | 0.70 |
| GalS | 0.22 | 0.56 | 0.22 | 0.20 | 0.67 |
| GcvA | 0 | 0 | 0 | 0 | 0 |
| GlpR | 0.09 | 0.22 | 0.04 | 0.17 | 0.30 |
| GntR | 0.24 | 0.29 | 0.24 | 0.27 | 0.59 |
| H-NS | 0.32 | 0.24 | 0.18 | 0.28 | 0.06 |
| IclR | 0 | 0.10 | 0.10 | 0.10 | 0.10 |
| IHF | 0.41 | 0.47 | 0.41 | 0.63 | 0.18 |
| IscR | 0 | 0 | 0 | 0 | 0.25 |
| LexA | 0.54 | 0.67 | 0.46 | 0.35 | 0.71 |
| Lrp | 0.31 | 0.30 | 0.30 | 0.56 | 0.30 |
| MalT | 0.20 | 0.30 | 0.20 | 0.34 | 0.55 |
| MarA | 0.13 | 0.13 | 0.06 | 0.06 | 0.06 |
| MelR | 0 | 0.25 | 0.25 | 0.14 | 0.50 |
| MetJ | 0.26 | 0.37 | 0.41 | 0.31 | 0.33 |
| MetR | 0 | 0 | 0 | 0.07 | 0.33 |
| ModE | 0 | 0.25 | 0 | 0.14 | 0.50 |
| Nac | 0 | 0 | 0.10 | 0.08 | 0.10 |
| NagC | 0.14 | 0.21 | 0 | 0.17 | 0.50 |
| NanR | 0.50 | 0.50 | 0.67 | 0.18 | 0.83 |
| NarL | 0.37 | 0.46 | 0.46 | 0.72 | 0.65 |
| NarP | 0.19 | 0.19 | 0.06 | 0.33 | 0.44 |
| NtrC | 0.41 | 0.50 | 0.41 | 0.36 | 0.73 |
| OmpR | 0.15 | 0.10 | 0.15 | 0.16 | 0.30 |
| OxyR | 0 | 0 | 0 | 0 | 0 |
| PhoB | 0.29 | 0.21 | 0.21 | 0.13 | 0.36 |
| PhoP | 0.50 | 0.50 | 0.41 | 0.31 | 0.55 |
| PspF | 0 | 0 | 0 | 0 | 0 |
| PurR | 0.17 | 0.39 | 0.11 | 0.23 | 0.39 |
| RcsAB | 0 | 0 | 0 | 0 | 0 |
| Rob | 0 | 0 | 0 | 0 | 0 |
| SoxS | 0 | 0.06 | 0.06 | 0.02 | 0 |
| TorR | 0.13 | 0.50 | 0 | 0.28 | 0.63 |
| TrpR | 0.20 | 0.20 | 0.1 | 0.19 | 0.70 |
| TyrR | 0.11 | 0.16 | 0 | 0.19 | 0.32 |
| UxuR | 0 | 0 | 0 | 0.10 | 0.60 |
