# Supplementary material for: Using Sequence-Specific Chemical and Structural Properties of DNA to Predict Transcription Factor Binding Sites
Source: PLoS Comput Biol. 2010 Nov 18;6(11):e1001007. doi: 10.1371/journal.pcbi.1001007 (PMC2987836; doi:10.1371/journal.pcbi.1001007)
Supplement: Table S5 — Computational results for 44 TFs documented in DPInteract. Here, the cutoff values for the BvH, Match, and MATRIX SEARCH methods were each set to the lowest scoring sequence in the training set from which a model for a TF binding site was built. This approach, which guarantees that positive examples used in training are correctly classified, is different from that described in the Methods section. For the QPMEME method, cutoff values are set to −1, and for the SiteSlueth method, cutoff values are set to 0. For each method, the training set size and the number of predicted binding sites are given. In each case, the number of hits is approximately the same as that reported in [6]. The cross-validation score V is given in parentheses. In cross-validation, the available positive examples are divided into a training set and a testing set, as described in the main text. Models are built based on the training set and tested using the remaining positive examples. Recall that each model (derived through any of the five methods that we consider here) is built to ensure that the binding sites in the training set are classified correctly; however, the testing examples withheld from training may not be predicted perfectly by a method. Although the QPMEME method usually predicts a lower number of binding sites compared to any of the other methods, its cross-validation score is relatively low in most cases. These results are not discussed in the main text. (0.18 MB DOC) [file pcbi.1001007.s007.doc]

**Table S5.** Computational results for 44 TFs documented in DPInteract. Here, the cutoff values for the BvH, Match, and MATRIX SEARCH methods were each set to the lowest scoring sequence in the training set from which a model for a TF binding site was built. This approach, which guarantees that positive examples used in training are correctly classified, is different from that described in the Methods section. For the QPMEME method, cutoff values are set to -1, and for the SiteSlueth method, cutoff values are set to 0. For each method, the training set size and the number of predicted binding sites are given. In each case, the number of hits is approximately the same as that reported in [6]. The cross-validation score *V* is given in parentheses. In cross-validation, the available positive examples are divided into a training set and a testing set, as described in the main text. Models are built based on the training set and tested using the remaining positive examples. Recall that each model (derived through any of the five methods that we consider here) is built to ensure that the binding sites in the training set are classified correctly; however, the testing examples withheld from training may not be predicted perfectly by a method. Although the QPMEME method usually predicts a lower number of binding sites compared to any of the other methods, its cross-validation score is relatively low in most cases. These results are not discussed in the main text.

| **Name** | **Training**  **Set Size** | **BvH** | **Match** | **MATRIX**  **SEARCH** | **QPMEME** | **Site**  **Sleuth** |
| --- | --- | --- | --- | --- | --- | --- |
| AraC | 6 | 5  (0.000) | 5  (0.050) | 5  (0.000) | 2  (0.000) | 5  (0.250) |
| ArcA | 13 | 495  (0.500) | 3094  (0.723) | 183  (0.385) | 79  (0.354) | 890  (0.608) |
| ArgR | 17 | 696  (0.609) | 17600  (0.774) | 327  (0.421) | 104  (0.335) | 1003  (0.618) |
| ArgR2 | 7 | 10  (0.071) | 12  (0.114) | 7  (0.050) | 6  (0.000) | 24  (0.257) |
| CpxR | 12 | 196  (0.354) | 559  (0.575) | 124  (0.296) | 65  (0.175) | 663  (0.542) |
| CRP | 49 | 9284  (0.860) | 188423  (0.948) | 7181  (0.738) | 1487  (0.490) | 3762  (0.706) |
| CytR | 5 | 455  (0.000) | 3322  (0.040) | 179  (0.000) | 105  (0.000) | 145  (0.000) |
| DnaA | 8 | 184724  (0.631) | 1030714  (0.569) | 18335  (0.488) | 1591  (0.063) | 273  (0.638) |
| FadR | 7 | 33  (0.107) | 43  (0.171) | 15  (0.071) | 14  (0.100) | 167  (0.329) |
| Fis | 19 | 9561  (0.176) | 610856  (0.661) | 9086  (0.137) | 799  (0.042) | 1322  (0.032) |
| FNR | 13 | 265  (0.396) | 1580  (0.642) | 81  (0.169) | 29  (0.138) | 475  (0.438) |
| FruR | 12 | 50  (0.317) | 195  (0.475) | 35  (0.221) | 30  (0.200) | 253  (0.642) |
| Fur | 9 | 344  (0.111) | 446  (0.072) | 119  (0.033) | 41  (0.000) | 335  (0.456) |
| GalR | 7 | 16  (0.043) | 22  (0.229) | 11  (0.007) | 5  (0.043) | 185  (0.486) |
| GlpR | 13 | 14322  (0.496) | 258867  (0.608) | 4514  (0.219) | 942  (0.131) | 709  (0.215) |
| H-NS | 15 | 24791  (0.420) | 51567  (0.587) | 21094  (0.350) | 7036  (0.153) | 7992  (0.087) |
| IHF | 26 | 122682  (0.504) | 438005  (0.794) | 89014  (0.413) | 684  (0.119) | 1358  (0.135) |
| LexA | 19 | 56  (0.505) | 476  (0.737) | 50  (0.266) | 31  (0.353) | 185  (0.837) |
| Lrp | 14 | 95064  (0.318) | 550985  (0.575) | 31655  (0.211) | 2865  (0.043) | 1783  (0.079) |
| MalT | 10 | 262  (0.355) | 1020  (0.550) | 276  (0.305) | 104  (0.350) | 1043  (0.810) |
| MetJ | 15 | 1576  (0.650) | 34796  (0.750) | 638  (0.393) | 130  (0.340) | 394  (0.660) |
| MetJ3 | 10 | 10  (0.210) | 21  (0.240) | 9  (0.085) | 4  (0.150) | 30  (0.870) |
| MetR | 8 | 461  (0.213) | 1949  (0.281) | 194  (0.056) | 80  (0.188) | 519  (0.225) |
| NagC | 6 | 22  (0.042) | 109  (0.092) | 12  (0.000) | 8  (0.050) | 101  (0.033) |
| NarL | 10 | 434  (0.380) | 538  (0.450) | 134  (0.285) | 32  (0.150) | 398  (0.470) |
| NarP | 8 | 5  (0.181) | 42  (0.356) | 6  (0.188) | 0  (0.100) | 143  (0.363) |
| NtrC | 5 | 4  (0.050) | 4  (0.020) | 4  (0.010) | 1  (0.000) | 40  (0.900) |
| OmpR | 9 | 3364  (0.144) | 3170  (0.122) | 1258  (0.000) | 197  (0.044) | 515  (0.100) |
| PhoB | 15 | 376  (0.517) | 1706  (0.580) | 171  (0.310) | 22  (0.200) | 409  (0.573) |
| PhoB3 | 5 | 4  (0.020) | 4  (0.060) | 4  (0.000) | 2  (0.000) | 7  (0.280) |
| PurR | 22 | 55  (0.425) | 480  (0.795) | 38  (0.323) | 31  (0.214) | 232  (0.695) |
| RpoD15 | 27 | 45582  (0.417) | 200614  0.780) | 48773  (0.302) | 1691  (0.122) | 3301  (0.070) |
| RpoD16 | 48 | 33138  (0.761) | 58839  (0.832) | 30837  (0.695) | 2250  (0.340) | 5122  (0.375) |
| RpoD17 | 116 | 277367  (0.969) | 399278  (0.979) | 284774  (0.956) | 13946  (0.568) | 17877  (0.536) |
| RpoD18 | 34 | 19666  (0.571) | 67058  (0.847) | 17445  (0.501) | 1043  (0.215) | 2686  (0.300) |
| RpoD19 | 25 | 20087  (0.316) | 93221  (0.722) | 17852  (0.250) | 1205  (0.088) | 1820  (0.104) |
| RpoH2 | 7 | 5  (0.000) | 325  (0.079) | 5  (0.000) | 4  (0.000) | 6  (0.000) |
| RpoH3 | 8 | 8  (0.000) | 5  (0.006) | 8  (0.000) | 8  (0.000) | 10  (0.225) |
| RpoN | 6 | 7  (0.117) | 16  (0.125) | 7  (0.133) | 6  (0.033) | 129  (0.417) |
| RpoS17 | 15 | 18993  (0.120) | 409478  (0.600) | 10935  (0.057) | 821  (0.020) | 527  (0.000) |
| RpoS18 | 7 | 578  (0.000) | 2537  (0.021) | 182  (0.000) | 28  (0.000) | 47  (0.000) |
| SoxS | 14 | 2761  (0.107) | 58423  (0.389) | 1633  (0.093) | 252  (0.050) | 227  (0.029) |
| Tus | 6 | 5  (0.250) | 6  (0.233) | 5  (0.133) | 6  (0.117) | 17  (0.950) |
| TyrR | 17 | 26996  (0.576) | 47269  (0.791) | 7043  (0.400) | 172  (0.182) | 696  (0.482) |
