# Supplementary material for: Using Sequence-Specific Chemical and Structural Properties of DNA to Predict Transcription Factor Binding Sites
Source: PLoS Comput Biol. 2010 Nov 18;6(11):e1001007. doi: 10.1371/journal.pcbi.1001007 (PMC2987836; doi:10.1371/journal.pcbi.1001007)
Supplement: Table S6 — Area under the curve (AUC) analysis for transcription factors (TFs) in RegulonDB with at least 20 known binding sites. A receiver operating characteristics (ROC) curve is a two-dimensional plot of the false positive rate (1 - specificity) versus the true positive rate (sensitivity). The AUC for an ROC curve is between 0 and 1. A perfect classifier will have an AUC of 1 and a random classifier will have an AUC of 0.5. We implemented an algorithm for generating ROC curves and for calculating AUCs, which allows us to rank classifiers. Positive examples in ReglonDB for a given TF are randomly divided into 2/3 positive training examples and 1/3 positive testing examples. The non-coding portions of the E. coli genome were used to generate all possible negative examples of TF binding sites. We built classifier models using the training examples for the five methods under consideration. The models are used to calculate scores for positive testing examples and negative examples. An ROC curve and the corresponding AUC were estimated from these scores for each model. For each TF, we performed the above procedure ten times to estimate ten AUCs for each method, and we report the average value and standard deviation of AUC in this table. (0.07 MB DOC) [file pcbi.1001007.s008.doc]

**Table S6.** Area under the curve (AUC) analysis for transcription factors (TFs) in RegulonDB with at least 20 known binding sites. A receiver operating characteristics (ROC) curve is a two-dimensional plot of the false positive rate (1 - specificity) versus the true positive rate (sensitivity). The AUC for an ROC curve is between 0 and 1. A perfect classifier will have an AUC of 1 and a random classifier will have an AUC of 0.5. We implemented an algorithm for generating ROC curves and for calculating AUCs, which allows us to rank classifiers. Positive examples in ReglonDB for a given TF are randomly divided into 2/3 positive training examples and 1/3 positive testing examples. The non-coding portions of the *E. coli* genome were used to generate all possible negative examples of TF binding sites. We built classifier models using the training examples for the five methods under consideration. The models are used to calculate scores for positive testing examples and negative examples. An ROC curve and the corresponding AUC were estimated from these scores for each model. For each TF, we performed the above procedure ten times to estimate ten AUCs for each method, and we report the average value and standard deviation of AUC in this table.

|  | SiteSlueth | BvH | Match | MATRIXSEARCH | QPMEME |
| --- | --- | --- | --- | --- | --- |
| AraC | 0.913±0.034 | 0.843±0.055 | 0.813±0.067 | 0.805±0.061 | 0.853±0.049 |
| ArcA | 0.870±0.022 | 0.926±0.013 | 0.913±0.019 | 0.926±0.013 | 0.918±0.017 |
| ArgR | 0.966±0.027 | 0.957±0.032 | 0.926±0.058 | 0.955±0.029 | 0.946±0.026 |
| CpxR | 0.886±0.050 | 0.919±0.041 | 0.895±0.042 | 0.913±0.046 | 0.856±0.059 |
| CRP | 0.947±0.015 | 0.925±0.015 | 0.900±0.020 | 0.926±0.015 | 0.899±0.013 |
| Fis | 0.787±0.029 | 0.805±0.047 | 0.798±0.041 | 0.807±0.046 | 0.770±0.025 |
| FlhDC | 0.892±0.040 | 0.767±0.052 | 0.728±0.060 | 0.762±0.057 | 0.704±0.087 |
| FNR | 0.951±0.038 | 0.964±0.026 | 0.953±0.029 | 0.963±0.026 | 0.952±0.020 |
| Fur | 0.960±0.023 | 0.933±0.027 | 0.900±0.041 | 0.921±0.030 | 0.935±0.014 |
| GlpR | 0.925±0.023 | 0.951±0.026 | 0.910±0.041 | 0.928±0.031 | 0.944±0.033 |
| H-NS | 0.677±0.097 | 0.836±0.037 | 0.802±0.058 | 0.819±0.047 | 0.776±0.039 |
| IHF | 0.858±0.020 | 0.894±0.019 | 0.876±0.023 | 0.893±0.019 | 0.846±0.020 |
| LexA | 0.938±0.044 | 0.923±0.049 | 0.908±0.072 | 0.916±0.050 | 0.918±0.039 |
| Lrp | 0.803±0.051 | 0.790±0.035 | 0.746±0.052 | 0.793±0.036 | 0.791±0.027 |
| MalT | 0.986±0.014 | 0.946±0.023 | 0.876±0.049 | 0.949±0.018 | 0.951±0.013 |
| MetJ | 0.772±0.067 | 0.678±0.050 | 0.665±0.058 | 0.693±0.049 | 0.657±0.053 |
| NarL | 0.917±0.033 | 0.836±0.026 | 0.796±0.046 | 0.839±0.025 | 0.817±0.020 |
| NtrC | 0.966±0.033 | 0.959±0.038 | 0.935±0.052 | 0.959±0.030 | 0.959±0.028 |
| OmpR | 0.944±0.026 | 0.938±0.031 | 0.918±0.042 | 0.902±0.042 | 0.949±0.021 |
| PhoP | 0.987±0.028 | 0.966±0.036 | 0.946±0.036 | 0.976±0.036 | 0.984±0.039 |
